# Effects of different protein sources on rumen fermentation-microbial characteristics, nutrient utilization, and fecal and urinary metabolites in periparturient breeding cows

**DOI:** 10.3389/fmicb.2026.1834901

**Published:** 2026-06-03

**Authors:** Yaqiong Ren, ChunFang Ma, JinBao Zhang, Lisha Ma, Yapeng Qin, Qiaoe Zhang

**Affiliations:** 1 College of Animal Science and Technology, Ningxia University, Yinchuan, China; 2 Ningxia Hui Autonomous Region Veterinary Drug and Feed Inspection Institute, Yinchuan, China; 3 Yongning County Animal Husbandry and Aquaculture Technology Extension Service Center Yongning, Yinchuan, China

**Keywords:** different protein sources, fecal and urinary metabolites, nutrient utilization, periparturient breeding cows, rumen fermentation-microbial characteristics

## Abstract

**Introduction:**

As a core nutrient regulating ruminant metabolic networks, immune homeostasis, and rumen microbial community structure, the source and supply of protein influence the adaptive capacity of breeding cows to metabolic stress during physiological transitions in the peripartum period.

**Methods:**

Sixty healthy, periparturient cows with similar body weights (573.14 ± 76.67 kg) and gestation lengths (228.77 ± 37.86 d) were selected and randomly divided into four groups (*n* = 15), each group received an isocaloric, isonitrogenous diet supplemented with soybean meal (Control Group, CG), rapeseed meal (Test Group 1, TG1), linseed cake (Test Group 2, TG2), or sunflower cake (Test Group 3, TG3) as the primary protein source. Rumen fermentation parameters, microbial composition and community structure, serum indicators, apparent nutrient digestibility, nitrogen utilization, and differential metabolites in feces and urine were measured to investigate the effects of different protein sources on rumen fermentation-microbial characteristics, nutrient utilization, and fecal/urinary metabolites in periparturient breeding cows.

**Results:**

(1) pH levels in the TG2 increased significantly at 30 days postpartum (PP30 d), peptidyltransferase (PLT) counts in the TG2 and TG3 were significantly higher than those in the CG and TG1 at calving day (0 d); Proteolytic enzymes (Pro), urease (URE), and PLT levels in the TG3 increased significantly at PP30 d; beneficial bacteria such as *Fibrobacter* and *Akkermansia* were significantly enriched in the TG2 and TG3 (*p* < 0.05). (2) In the TG3, Urea and triglycerides (TG) levels were significantly elevated at PP30 d, while blood ammonia levels were reduced; in the TG2, energy metabolism was active at 0 d and stabilized at PP30 d, exhibiting the strongest humoral immune response and significantly elevated Gonadotropin-releasing hormone (GnRH) levels. The TG3 played a prominent role in the phasic regulation of insulin (INS) and growth hormone (GH). (3) Compared with the CG, 498, 285, and 225 differentially expressed metabolites were identified in the feces of the experimental groups, respectively; the TG1 was enriched in steroids, riboflavin, and the NF-κB pathway; the TG2 was enriched in riboflavin, steroids, retinol, and alkaloid pathways; and the TG3 showed a regulatory trend toward bile secretion and steroid synthesis; A total of 259, 456, and 234 differentially expressed metabolites were identified in urine, respectively. The TG1 primarily affected protein and carbohydrate metabolism; the TG2 focused on amino acid metabolism and biosynthesis; and the TG3 showed significant enrichment in the biosynthesis of unsaturated fatty acids, biotin metabolism, and folate biosynthesis pathways.

**Discussion:**

The rapeseed meal group caused excessive protein fermentation, leading to the production of endogenous mycotoxins and triggering oxidative stress and metabolic burden. The sunflower meal group exhibited elevated levels of intestinal toxins and permeability markers, which impaired reproductive performance. The linseed cake group maintains ruminal fermentation and intestinal metabolism in cows, making it a high-quality protein source for feeding periparturient breeding cows as a substitute for soybean meal.

## Graphical abstract

**Figure:**
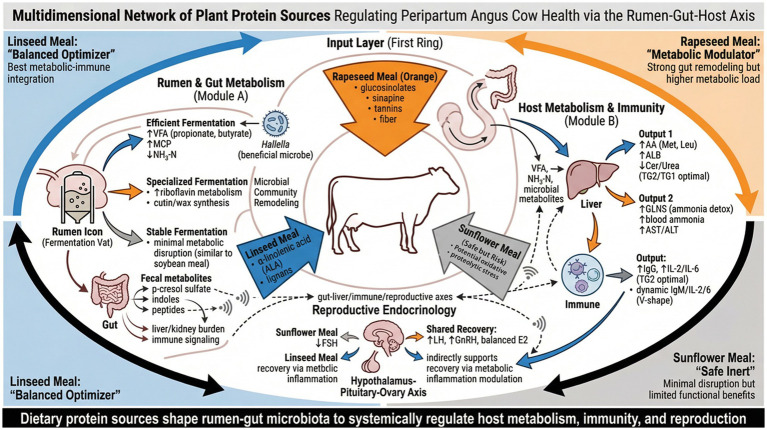

## 1 Introduction

Nutritional regulation during the peripartum period has a direct and critical impact on cow health and calf survival (Shang et al., 2024; Cao et al., 2019). As a core nutrient regulating ruminant metabolic networks, immune homeostasis, and rumen microbial community structure, the source and supply of protein influence the adaptive capacity of breeding cows to metabolic stress during physiological transitions in the peripartum period. It serves as a key regulatory target for ensuring cow homeostasis and production performance (Costa, 2022; Ziegler, 2025). Nutrient utilization and rumen homeostasis during the peripartum period of breeding cows directly determine their postpartum recovery and reproductive performance. As the core digestive organ, rumen function is significantly regulated by dietary protein sources. Differences in degradation rates, Amino Acids (AA) composition, and fermentation efficiency among various protein sources reshape rumen microbial community structure and fermentation patterns (McCann et al., 2017; Morales et al., 2024). Not only does it affect the levels of volatile fatty acids (VFA) and microbial crude protein (MCP), but it also ultimately impacts the nutritional status and production performance of cows by altering the overall digestion and absorption of nutrients. The systemic metabolism of periparturient breeding cows is highly dependent on protein source supply. Appropriate protein sources can alleviate metabolic stress, enhance immune and antioxidant functions by optimizing serum AA levels. Conversely, inappropriate protein sources may disrupt rumen homeostasis, trigger metabolic disorders, and significantly increase the risk of postpartum metabolic diseases (Meneses et al., 2024; Coleman et al., 2020). However, it remains unclear how different protein sources specifically regulate nutrient utilization and rumen function in reproductive cows during the periparturient period. Furthermore, most current studies analyze metabolic profiles at a single time point and have not yet effectively established a link between characteristics on the day of calving and subsequent health recovery patterns during the periparturient period. Soybean meal serves as a classic high-quality protein source for ruminants, offering advantages such as balanced AA profiles, suitable degradation characteristics, and good palatability. It effectively meets the essential AA requirements of periparturient breeding cows and is therefore frequently used as a benchmark for evaluating other protein sources (Gonçalves et al., 2025). Rapeseed meal and sunflower cake, with their abundant resources, low cost, and substantial protein content, have become important alternatives in livestock production for reducing dependence on soybean meal and controlling farming costs (Xie et al., 2025; Zhu et al., 2006). Linseed cake is rich in protein, dietary fiber, and alpha-linolenic acid, playing a positive role in enhancing production performance and improving milk and meat quality (Fang et al., 2024; Tao et al., 2022). Although previous studies have examined the effects of rapeseed meal, linseed cake, and sunflower cake in ruminant diets, most of these studies have significant limitations. The research stage mainly focused on the lactation period or fattening period, but lacked systematic exploration of the critical metabolic stress window during the perinatal period. Furthermore, most studies have compared a single protein source with soybean meal, lacking direct comparisons of the three protein sources under the same experimental conditions. Additionally, evaluation metrics have largely centered on production performance, with insufficient comprehensive assessment of nutrient utilization, nitrogen metabolic efficiency, and markers of metabolic stress. Therefore, there remains a lack of systematic evaluation regarding whether the use of rapeseed meal, linseed cake, and sunflower cake in the diets of breeding cows during the periparturient period affects nutrient utilization or exacerbates metabolic stress in cows (Zhou et al., 2016), and their relative merits and specific effects on reproductive performance remain unclear. This study used periparturient breeding cows as the research subjects and soybean meal as the control to evaluate the feed value of rapeseed meal, linseed cake, and sunflower cake as protein sources. By analyzing differences in microbial composition, microbial community structure, and fecal and urinary metabolites on the day of calving (0 d), combined with the detection of rumen fermentation parameters, serum indicators, apparent digestibility of nutrients, and nitrogen utilization rates (NUR) at 0 d, 15 days postpartum (PP15 d), and PP30 d, reveals the effects of different protein sources on the rumen fermentation-microbial characteristics, nutrient utilization, and fecal and urine metabolites of cows. It clarifies the mechanisms and feasibility of replacing soybean meal with rapeseed meal, flax cake, and sunflower cake in the diet of cows during this physiological stage, providing a theoretical basis for designing the diet formula for these cows at this stage. We assume the following: (1) The apparent digestibility of nutrients from the three protein sources may be similar to that of soybean meal; (2) Because linseed cake is rich in *α*-linolenic acid, its effect in alleviating metabolic stress may be superior to that of rapeseed meal and sunflower cake; (3) The effects of rapeseed meal and sunflower cake on the metabolic burden, oxidative stress and reproductive performance of lactating cows may differ from those of soybean meal.

## 2 Materials and methods

### 2.1 Experimental materials

Soybean meal, rapeseed meal, linseed cake, and sunflower cake used in the experiment were provided by Ningxia Benwang Ecological Agriculture Co., Ltd.

The processing methods and nutritional composition of the four protein ingredients (soybean meal, rapeseed meal, linseed cake, and sunflower cake) are presented in Supplementary Table S1. Soybean meal and rapeseed meal were measured in our laboratory. Linseed cake data from China Feed Database (No. 5-10-058, Ningxia Qingtongxia, pressed). Sunflower cake data from Heatland Lysine, Inc. (1988), consistent with recent literature (Shi et al., 2025).

### 2.2 Experimental animals and design

Sixty healthy Angus cows were used in this study. At the start of the trial, the overall body weight was 573.14 ± 76.67 kg and the gestation length was 228.77 ± 37.86 d. Cows were randomly allocated to 4 groups of 15 animals each. The protein sources in the diets for each group were soybean meal (CG), rapeseed meal (TG1), linseed cake (TG2), and sunflower cake (TG3). The trial included 7d pre-trial period and 120 d main trial period (90 d before calving to 30 d after calving). During the trial, all groups had free access to feed, water, and movement. Metagenomic and metabolomic analyses were performed exclusively on rumen fluid, fecal, and urine samples collected on 0 d to obtain high-resolution microbial and metabolic profiles on the day of calving, which were used for subsequent association analyses of postpartum recovery phenotypes. Concurrently, serum samples were collected at 0 d, PP15 d, and PP30 d to measure serum markers, apparent nutrient digestibility, nitrogen utilization, and other indicators, thereby systematically mapping the trajectory of postpartum physiological recovery.

### 2.3 Experimental diets

The daily ration formulation is based on the NRC (2016) Nutrient Requirements of Beef Cattle. The primary ingredient composition and nutritional levels are shown in Table 1.

**Table 1:** Experimental daily ration formulation and nutritional levels (DM, %).

Composition	CG	TG1	TG2	TG3
Corn silage	33.00	42.05	41.92	33.01
Rice straw	19.35	19.18	19.12	19.36
Corn cob pellets	21.33	14.92	14.87	18.82
Corn grain (ground)	10.91	7.21	7.19	10.91
Soybean Meal	9.44	0.00	0.00	0.00
Rapeseed Meal	0.00	9.95	0.00	0.00
Linseed cake	0.00	0.00	10.24	0.00
Sunflower Cake	0.00	0.00	0.00	11.15
Wine Pomace	2.73	3.48	3.47	3.51
5% Cattle Compound Premix^1^	1.71	1.69	1.69	1.71
Expanded Urea	0.42	0.41	0.41	0.42
Sodium bicarbonate	0.14	0.14	0.14	0.14
Sodium chloride	0.56	0.55	0.55	0.56
Magnesium Oxide	0.06	0.06	0.06	0.06
Dicalcium Phosphate	0.28	0.28	0.28	0.28
Compound Enzyme Preparation	0.08	0.08	0.08	0.08
Total	100.00	100.00	100.00	100.00
Nutritional level				
NEL (Mcal/kg)^2^	1.57	1.55	1.55	1.55
Starch	20.50	19.90	20.30	20.20
CP	12.00	12.00	11.80	11.90
MP	8.53	8.30	8.30	8.47
NDF	42.40	41.80	41.80	40.70
ADF	30.40	30.80	30.70	31.00
Lys (% of MP)	7.24	7.33	7.19	7.07
Met (%of MP)	2.36	2.38	2.43	2.36
Ca	0.36	0.38	0.36	0.36
P	0.32	0.35	0.32	0.36

^1^Per kilogram of premix provides: Fe 1,500 mg, Mn 1,000 mg, Se 12 mg, Cu 350 mg, Zn 1700 mg, I 7 mg, Co 10 mg, VE 1800 mg, VA 2 × 10^5^ IU, VD 8 × 10^4^ IU. ^2^NEL, MP, Lys (% of MP), and Met (% of MP) were calculated values; all other items were measured values. NEL, net energy for lactation; CP, crude protein; MP, metabolizable protein; NDF, neutral detergent fiber; ADF, acid detergent fiber; Lys, lysine; Met, methionine; Ca, calcium; P, phosphorus.

### 2.4 Sample collection and processing

#### 2.4.1 Diet

Dietary samples were collected from cows at 0 d, PP15 d, and PP30 d to determine dry matter (DM), crude protein (CP), acid detergent fiber (ADF), neutral detergent fiber (NDF), acid-insoluble ash (AIA), and starch content.

#### 2.4.2 Rumen fluid

Ruminal fluid was collected 3 h after morning feeding at 0 d, PP15 d, and PP30 d. After rapid pH measurement, a portion of the ruminal fluid was stored at - 20 °C for determination of ruminal fermentation parameters. A separate portion of the ruminal fluid collected at 0 d was stored at - 80 °C for analysis of microbial composition and community structure.

#### 2.4.3 Blood samples

Blood samples (20 mL) were collected from the jugular vein of cows at 0 d, PP15 d, and PP30 d before morning feeding. Samples were refrigerated at 4 °C for 1 h, then centrifuged at 3000 rpm for 10 min for serum parameter analysis.

#### 2.4.4 Fecal samples

Fecal samples were collected from cows at 0 d, PP15 d, and PP30 d and divided into two portions. One portion was treated with 10% sulfuric acid for nitrogen fixation to determine fecal nitrogen content; the other portion was not treated for nitrogen fixation and was used to DM and AIA content. Fecal samples collected at 0 d were reserved for metabolomics analysis.

#### 2.4.5 Urine samples

Urine samples were collected from cows at 0 d, PP15 d and PP30 d and divided into two portions. One portion was treated with 10% sulfuric acid at a 1:1 ratio for nitrogen fixation and subsequently used to determine urinary nitrogen content. The other portion, untreated for nitrogen fixation, was stored at −80 °C for creatinine (Cre) measurement. Urine collected at 0 d was reserved for metabolomics analysis.

### 2.5 Determination indicators and methods

DM and AIA: National Standard Methods (GB/T 6435–2014 and GB/T 23742–2009).

CP: Kjeldahl Nitrogen Determination Method (GB/T 6432–2018).

Fecal Output (kg/d) = AIA Intake (kg/d) / AIA Content in Feces (kg/kg).

Urine Output (L/d) = Body Weight (kg) × 29 / Creatinine Content (mg/L).

Nitrogen digestibility (%) = (Nitrogen intake - Fecal nitrogen excretion) / Nitrogen intake × 100.

Nitrogen utilization rate (%) = (Nitrogen intake - Fecal nitrogen excretion - Urinary nitrogen excretion) / Nitrogen intake × 100.

Apparent digestibility (%) = [(Nutrient intake - Fecal nutrient output) / Nutrient intake] × 100. This equation was used to determine the digestibility of dry matter (DMD), crude protein (CPD), neutral detergent fiber (NDFD), and acid detergent fiber (ADFD).

Rumen fluid pH was measured using a pH meter; Ammonia nitrogen (NH₃-N) was determined by the phenol-sodium hypochlorite colorimetric method; Rumen MCP was determined using the Coomassie Brilliant Blue method; VFA was determined using an Agilent 7820A gas chromatograph equipped with a flame ionization detector and a DB-FFAP capillary column (30 m × 250 μm × 0.25 μm, Agilent 122–3,232). The injector temperature was set to 220 °C, and the split ratio was 30:1. Detector temperature was 220 °C, with an air flow rate of 400 mL/min, a hydrogen flow rate of 40 mL/min, and a purge gas flow rate of 25 mL/min. The carrier gas was nitrogen, and the column flow rate was 1.06 mL/min. The column oven program was as follows: hold at 80 °C for 2 min, then ramp at 10 °C/min to 200 °C, and hold for 17 min; Pro, PLT, URE, lactate dehydrogenase (LDH) and peptidase (PEP) in rumen fluid were detected via ELISA.

Key Enzymes in Serum Protein Metabolism: Glutamine synthetase (GLNS), glutamine synthetase (GS), creatine kinase (CK), alanine aminotransferase (ALT), and aspartate aminotransferase (AST).

Nitrogen metabolism indicators: Cre, Urea, total protein (TP), albumin (ALB), globulin (GLB), and blood Ammonia.

Immune and inflammatory markers: Immunoglobulin A (IgA), immunoglobulin G (IgG), immunoglobulin M (IgM), interleukin-2 (IL-2), interleukin-6 (IL-6), and tumor necrosis factor-*α* (TNF-α).

Serum biochemical indicators: TG, Glucose (GLU), non-esterified Fatty Acids (NEFA), vitamin A (VA), vitamin D (VD), vitamin E (VE), vitamin K (VK) and *β*-hydroxybutyrate (BHBA).

Reproductive hormones: GLB, GH, GnRH, progesterone (E), luteinizing hormone (LH), INS, progesterone (PROG) and follicle-stimulating hormone (FSH).

Cre, Urea, TP, ALB, Blood ammonia, GLU, and TG were measured using biochemical assay kits, while the remaining serum parameters were analyzed via ELISA.

### 2.6 Omics analysis

#### 2.6.1 Metagenomics

1. Metagenomic sequencing of rumen fluid was performed using the Illumina NovaSeq™ X Plus platform.
2. Raw data quality control: Adapter sequences were removed using fastp software, and reads shorter than 50 bp or with an average quality below 20 bp were filtered out. The remaining high-quality reads were aligned against the bovine genome using BWA software, and contaminated sequences were removed.
3. The quality-controlled high-quality data were assembled using MEGAHIT to obtain contigs with a length of at least 300 bp.
4. Gene prediction was performed using Prodigal. CD-HIT constructed a non-redundant gene set, and SOAPaligner calculated gene abundance.
5. The non-redundant gene set underwent species and functional annotation by aligning it against databases such as NR and KEGG using DIAMOND.

#### 2.6.2 Fecal and urinary metabolomics

Fecal and urine metabolomics analyses were performed using LC–MS technology, and quality control (QC) samples were interspersed to monitor analytical stability. Progenesis QI software was used for raw data preprocessing and metabolite identification against databases. For data quality control, the 80% rule was applied to filter missing values, followed by sum normalization, and variables with RSD > 30% in QC samples were removed. Differential metabolites were screened using VIP > 1 and *p* < 0.05 as criteria, followed by KEGG pathway annotation and enrichment analysis.

### 2.7 Data processing and statistical analysis

Experimental data were processed using Excel 2019 and subjected to repeated measures analysis of variance (ANOVA) with SAS 9.4. Pairwise comparisons between groups underwent Bonferroni correction. *p* < 0.05 indicated significant differences, while *p* < 0.01 denoted highly significant differences. Results are expressed as mean ± standard deviation.

First, compared omics enrichment results at the pathway level. Subsequently, calculated Spearman correlation coefficients between multi-omics data and rumen fermentation parameters as well as key serum indicators for integrated analysis.

## 3 Results

### 3.1 Rumen fermentation - microbial characteristics

#### 3.1.1 Rumen fermentation parameters and protein utilization enzymes

As shown in Tables 2, 3, the pH level in the TG2 at PP30 d was significantly higher than at PP15 d (*p* < 0.05). The propionate content in the TG1 at PP30 d was significantly higher than at 0 d (*p* < 0.05). The isobutyrate level in the CG at PP15 d was significantly lower than at 0 d (*p* < 0.05). No significant differences were observed among groups at other time points.

**Table 2:** Rumen fermentation parameters.

Item	Days postpartum	CG	TG1	TG2	TG3	*F*	*p*
pH	0 d	7.14 ± 0.31	7.25 ± 0.31	7.05 ± 0.61	6.90 ± 0.41	1.132	0.350
	15 d	6.88 ± 0.41	6.99 ± 0.21	6.91 ± 0.21	7.05 ± 0.41	0.461	0.711
	30 d	6.87 ± 0.41	7.01 ± 0.31	7.30 ± 0.61^b^	6.94 ± 0.21	1.737	0.178
	F	1.960	1.571	3.894	0.376		
	*p*	0.157	0.223	0.030	0.689		
NH_3_-N (mg/100 mL)	0 d	7.82 ± 3.23	5.43 ± 3.01	6.97 ± 2.76	6.53 ± 2.90	1.145	0.344
	15 d	6.91 ± 1.94	6.80 ± 2.35	5.70 ± 2.07	6.79 ± 2.17	0.671	0.575
	30 d	8.85 ± 2.14	6.60 ± 2.26	6.27 ± 1.78	8.43 ± 2.05	3.857	0.017
	F	1.934	0.626	0.431	1.879		
	*p*	0.160	0.541	0.653	0.168		
MCP (g/L)	0 d	0.54 ± 0.22	0.51 ± 0.26	0.69 ± 0.31	0.58 ± 0.16	0.976	0.084
	15 d	0.65 ± 0.27	0.44 ± 0.19	0.62 ± 0.20	0.65 ± 0.16	1.860	0.148
	30 d	0.44 ± 0.12	0.38 ± 0.15	0.58 ± 0.33	0.49 ± 0.09	0.190	0.136
	F	2.808	0.729	9.543	1.582		
	*p*	0.076	0.491	0.586	0.222		
Acetate (mmol/L)	0 d	58.75 ± 16.56	41.41 ± 11.12	47.22 ± 17.54	41.27 ± 15.88	2.814	0.053
	15 d	58.24 ± 10.41	54.76 ± 20.18	59.73 ± 8.78	53.85 ± 15.29	0.378	0.770
	30 d	63.02 ± 11.58	59.71 ± 12.62	63.94 ± 26.32	53.62 ± 23.78	0.562	0.643
	F	0.205	3.381	2.890	2.301		
	*p*	0.816	0.045	0.069	0.115		
Propionate (mmol/L)	0 d	15.04 ± 4.30	11.24 ± 3.00	14.05 ± 4.36	11.32 ± 3.93	2.394	0.084
	15 d	15.57 ± 2.60	14.89 ± 5.22	16.94 ± 2.61	14.09 ± 4.34	0.976	0.415
	30 d	17.24 ± 2.76	17.13 ± 3.82^a^	17.56 ± 7.01	14.58 ± 6.64	0.655	0.585
	F	0.502	4.619	2.213	1.988		
	*p*	0.610	0.017	0.124	0.152		
Butyrate (mmol/L)	0 d	7.98 ± 2.30	6.16 ± 1.54	6.51 ± 2.52	5.98 ± 2.24	1.738	0.177
	15 d	8.74 ± 1.49	7.72 ± 2.64	7.91 ± 1.28	8.04 ± 2.99	0.396	0.757
	30 d	9.06 ± 2.09	8.05 ± 2.36	8.69 ± 3.36	6.85 ± 2.89	1.266	0.301
	F	0.580	2.039	2.271	2.258		
	*p*	0.565	0.145	0.118	0.120		
Isobutyrate (mmol/L)	0 d	1.23 ± 0.31	1.00 ± 0.21	0.98 ± 0.33	0.93 ± 0.25	2.266	0.097
	15 d	0.99 ± 0.10^a^	0.94 ± 0.20	1.01 ± 0.14	0.99 ± 0.16	0.353	0.787
	30 d	1.06 ± 0.08	1.04 ± 0.21	0.98 ± 0.24	0.92 ± 0.35	0.685	0.567
	F	4.267	0.669	0.104	0.454		
	*p*	0.022	0.518	0.901	0.639		
Valerate (mmol/L)	0 d	1.37 ± 0.40	1.04 ± 0.27	1.13 ± 0.32	1.10 ± 0.25	2.150	0.111
	15 d	1.40 ± 0.22	1.25 ± 0.36	1.37 ± 0.30	1.23 ± 0.33	0.745	0.532
	30 d	1.48 ± 0.27	1.40 ± 0.32	1.37 ± 0.45	1.21 ± 0.49	0.874	0.463
	F	0.245	3.303	2.612	0.673		
	*p*	0.784	0.049	0.088	0.517		
Isovalerate (mmol/L)	0 d	1.79 ± 0.54	1.26 ± 0.32	1.29 ± 0.52	1.27 ± 0.41	3.172	0.036
	15 d	1.44 ± 0.24	1.37 ± 0.48	1.45 ± 0.54	1.17 ± 0.15	1.085	0.368
	30 d	1.58 ± 0.26	1.41 ± 0.34	1.41 ± 0.43	1.23 ± 0.52	1.297	0.290
	F	2.574	0.409	0.583	0.189		
	*p*	0.091	0.667	0.563	0.829		
TVFA (mmol/L)	0 d	86.16 ± 23.83	62.11 ± 15.92	71.17 ± 25.27	61.87 ± 22.64	2.639	0.064
	15 d	86.37 ± 14.41	80.92 ± 28.72	88.42 ± 12.90	79.37 ± 22.25	0.439	0.727
	30 d	93.44 ± 16.72	88.75 ± 18.76	93.95 ± 37.38	78.41 ± 34.44	0.647	0.590
	F	0.235	3.453	2.695	2.183		
	*p*	0.792	0.043	0.082	0.128		

^a^indicates comparison with 0 d, *p* < 0.05; b indicates comparison with 15 d, *p* < 0.05; A indicates comparison with CG, *p* < 0.05; ^b^indicates comparison with TG1, *p* < 0.05; ^c^indicates comparison with TG2, *p* < 0.05. All pairwise comparisons underwent Bonferroni correction, as below.

**Table 3:** Overall test of rumen fermentation parameters.

Item	F_group_	F_Time_	F_Time × group_	P_group_	P_Time_	P_Time × group_
pH	0.536	1.320	1.966	0.661	0.274	0.083
NH_3_-N	4.391	1.661	0.862	0.197	0.527	0.010
MCP	3.177	3.208	0.483	0.037	0.047	0.819
Acetate	2.166	6.819	0.573	0.109	0.002	0.751
Propionate	2.565	7.495	0.430	0.070	0.002	0.832
Butyrate	2.546	5.146	0.421	0.071	0.008	0.862
Isobutyrate	1.664	0.687	1.213	0.192	0.506	0.310
Valerate	2.257	4.307	0.496	0.098	0.017	0.809
Isovalerate	3.005	0.228	1.052	0.043	0.797	0.400
TVFA	2.299	6.618	0.508	0.094	0.002	0.800

As shown in Tables 4, 5, the TG3 exhibited significantly higher Pro levels at PP30 d compared to 0 d and PP15 d, and significantly higher than the CG (*p* < 0.05). The TG2 demonstrated significantly higher URE levels at PP30 d compared to 0 d. Both the TG1 and TG3 showed significantly higher URE levels at PP30 d compared to 0 d and PP15 d, with the TG3 at PP30 d level significantly exceeding that of the CG (*p* < 0.05). The PLT at 0 d in the TG2 and TG3 was significantly higher than that in the CG and TG1. The PLT at PP30 d in the TG3 was significantly higher than that in the CG. The PLT at 0 d in the CG and TG1 was significantly lower than that at PP15 d and PP30 d. The PLT at PP30 d in the TG2 was significantly higher than that at PP15 d, and the PLT at PP30 d in the TG3 was significantly higher than that at 0 d and PP15 d. The PEP at PP30 d in the CG was significantly lower than PP15 d (*p* < 0.05).

**Table 4:** Protein utilization enzymes.

Item	Days postpartum	CG	TG1	TG2	TG3	F	*p*
Pro (μg/L)	0 d	0.87 ± 0.10	0.70 ± 0.12	0.87 ± 0.17	0.71 ± 0.09	3.862	0.022
	15 d	0.78 ± 0.16	0.72 ± 0.16	0.83 ± 0.09	0.75 ± 0.17	0.751	0.533
	30 d	0.65 ± 0.13	0.87 ± 0.12	0.86 ± 0.15	1.24 ± 0.49 ^Aab^	5.741	0.004
	F	1.592	1.369	0.099	14.076		
	*p*	0.225	0.274	0.907	<0.01		
LDH (U/L)	0 d	98.26 ± 46.52	117.31 ± 80.12	69.34 ± 47.82	85.79 ± 7.60	1.018	0.402
	15 d	134.92 ± 54.75	104.09 ± 68.22	135.19 ± 41.71	142.33 ± 11.92	0.783	0.515
	30 d	146.25 ± 30.92	116.17 ± 76.49	142.32 ± 76.81	141.35 ± 25.67	0.305	0.822
	F	1.078	0.111	2.782	1.815		
	*p*	0.357	0.896	0.083	0.185		
URE (ng/L)	0 d	188.90 ± 22.95	168.78 ± 23.99	174.28 ± 28.02	172.07 ± 12.91	1.077	0.378
	15 d	189.22 ± 30.18	186.01 ± 14.82	202.75 ± 20.27	194.81 ± 30.00	0.616	0.611
	30 d	195.72 ± 24.53	207.26 ± 19.68^ab^	211.49 ± 26.63^a^	242.80 ± 30.95^Aab^	4.270	0.015
	F	0.426	7.005	4.757	28.97		
	*p*	0.658	0.004	0.019	<0.001		
PLT (ng/L)	0 d	101.78 ± 22.29	123.35 ± 17.34	182.18 ± 24.48^AB^	167.06 ± 13.43^AB^	24.846	<0.01
	15 d	180.88 ± 23.46^a^	173.12 ± 41.45^a^	160.79 ± 22.95	162.28 ± 21.57	0.774	0.520
	30 d	178.60 ± 21.52^a^	194.72 ± 18.97^a^	195.58 ± 22.41^b^	223.74 ± 19.33^Aab^	5.796	0.004
	F	25.033	18.586	3.260	15.882		
	*p*	<0.001	<0.001	0.057	<0.001		
PEP (ng/mL)	0 d	5.29 ± 0.41	4.83 ± 0.39	5.20 ± 0.82	4.91 ± 0.43	1.158	0.346
	15 d	5.36 ± 0.92	4.73 ± 0.48	4.70 ± 0.58	4.75 ± 0.36	1.818	0.171
	30 d	4.47 ± 0.82^b^	4.81 ± 0.32	4.98 ± 0.78	5.48 ± 0.85	2.366	0.096
	F	3.793	0.073	1.601	2.624		
	*p*	0.038	0.930	0.223	0.094		

Note: a indicates comparison with 0 d, *p* < 0.05 b indicates comparison with 15 d, *p* < 0.05; A indicates comparison with CG, *p* < 0.05; B indicates comparison with TG1, *p* < 0.05; C indicates comparison with TG2, *p* < 0.05.

**Table 5:** Comprehensive assessment of protein utilization enzymes.

Item	F_group_	F_Time_	F_Time × group_	P_group_	P_Time_	P_Time × group_
Pro	2.151	4.567	5.950	0.120	0.015	<0.001
LDH	0.330	4.362	0.755	0.804	0.018	0.706
URE	0.984	26.154	3.527	0.417	<0.001	0.011
PLT	9.509	34.984	7.285	<0.001	<0.001	<0.001
PEP	0.722	0.697	2.138	0.549	0.508	0.066

#### 3.1.2 Microbial composition and microbiome structure

The metagenomic analyses presented in this section were based solely on rumen fluid samples collected on 0 d. These 0 d microbial profiles were subsequently used for correlation analysis with longitudinal parameters measured on 0 d, PP15 d and PP30 d (see Section 3.4).

**Figure 1:**
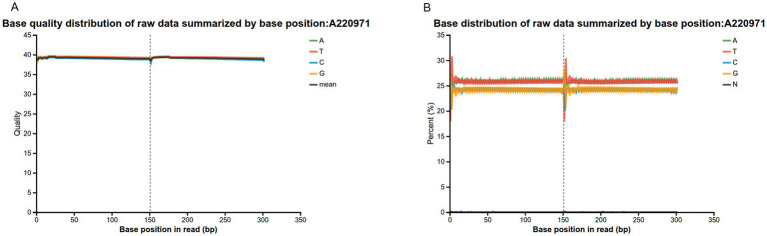
Sequence quality assessment of raw data. **(A)** Base quality per position for sample A220971. **(B)** Base composition per position. Quality profiles were consistent across all samples.

The average quality line remains significantly above Q30 across the entire read length range, exhibiting a smooth overall trend, indicating high sequencing data quality and accurate base calling (Figure 1A). Except for normal fluctuations in the initial 10–15 bases, the A, T, C, and G lines remained nearly parallel and closely overlapping throughout the subsequent read length, each approaching 25% (Figure 1B).

**Figure 2:**
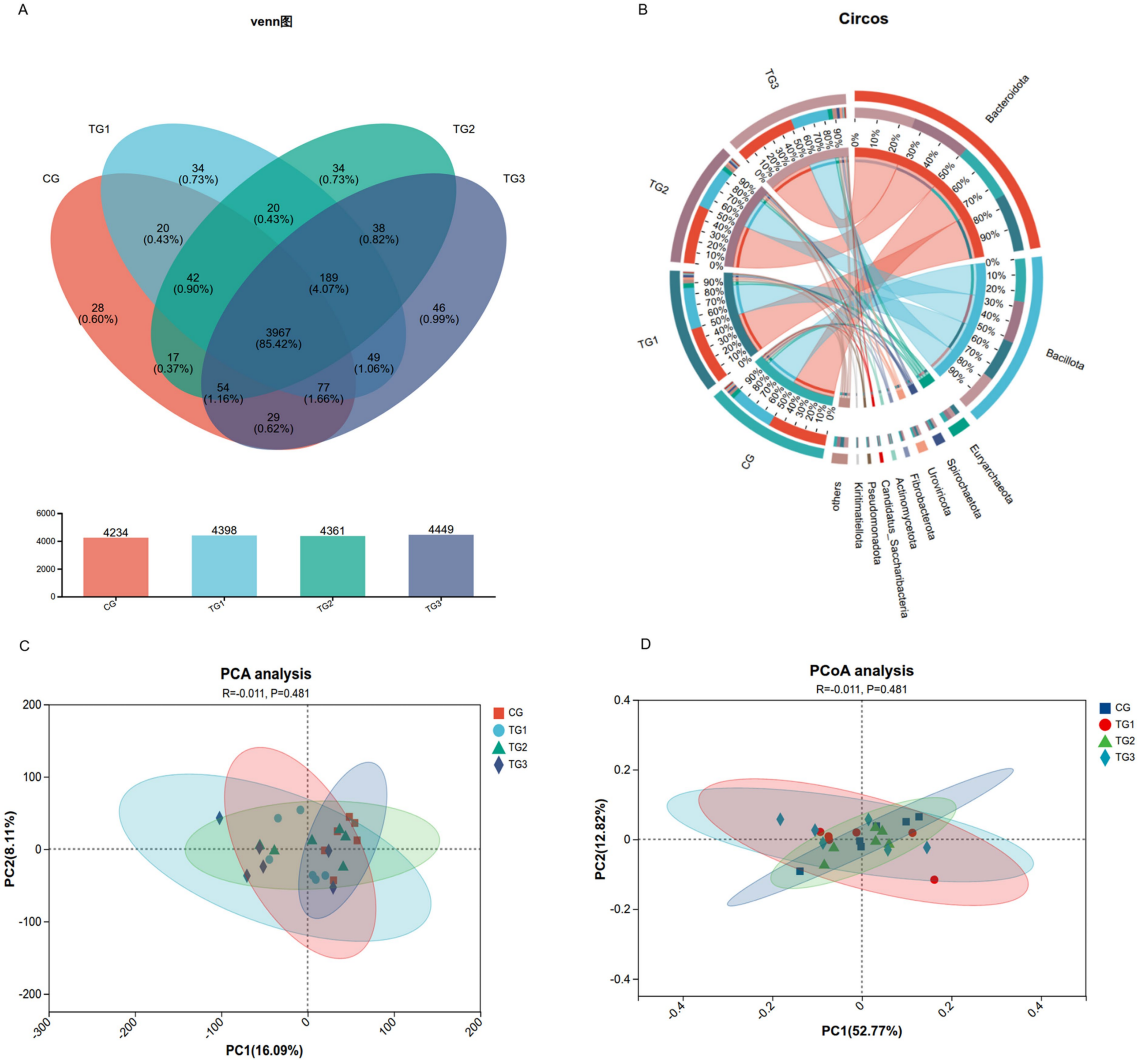
Multidimensional analysis of microbial community structure. **(A)** Venn diagrams showing shared and unique taxa among groups. **(B)** Circos plot displaying sample–species relationships. **(C)** Principal component analysis (PCA). **(D)** Principal coordinate analysis (PCoA).

NR species annotation indicates that the overlapping region among the four groups contains 3,967 shared species (85.42%), while each group harbors unique species: CG: 17 species (0.37%), TG1: 28 species (0.60%), TG2: 34 species (0.73%), TG3: 46 species (0.99%). These proportions were all relatively low, indicating a high overall consistency in microbial composition (Figure 2A). *Bacteroidota* are microorganisms primarily responsible for degrading complex carbohydrates, accounting for 26% in the TG3 and 25% in the other groups. *Bacillota* comprise various cellulose-degrading bacteria capable of secreting cellulase and hemicellulase to utilize cellulose and hemicellulose for energy production, representing 27, 25, 26, and 23% across the four groups, respectively. The dominant microbial groups in the CG and TG2 were *Bacillota*, while *Bacteroidota* dominated the TG3. The microbial composition of the TG1 fell between that of TG2 and TG3 (Figure 2B). PCA and PCoA analysis showed that samples from all groups clustered together, with no clear separation between groups (Figures 2C,D).

**Figure 3:**
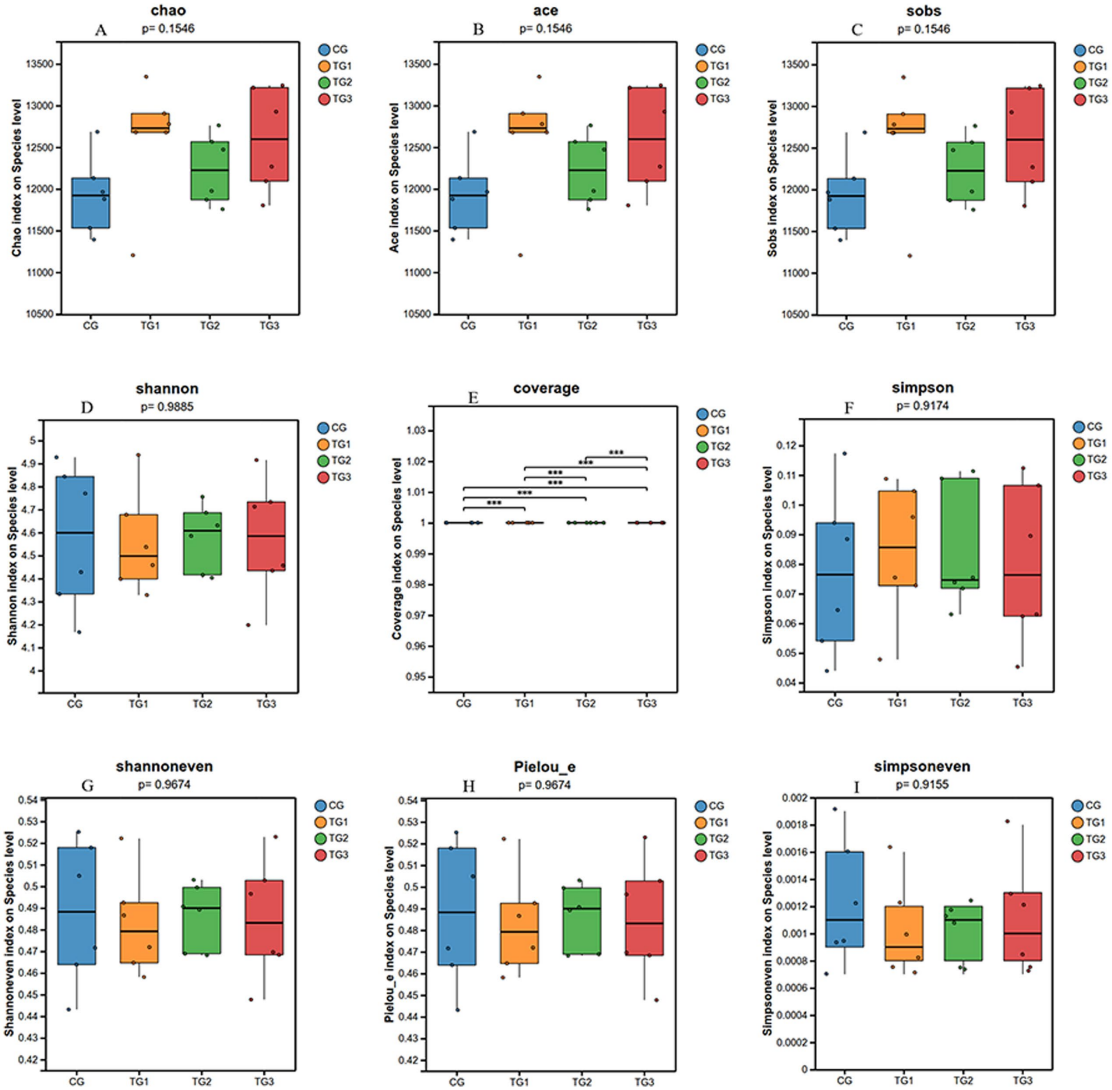
Alpha diversity indices of the microbial communities.Boxplots showing the distribution of nine alpha diversity metrics across all samples: **(A)** sobs; **(B)** ace; **(C)** chao; **(D)** shannon; **(E)** coverage; **(F)** simpson; **(G)** shannoneven; **(H)** Pielou_e; **(I)** simpsoneven.

There were no significant differences in microbial relative abundance among groups as measured by sobs, ace and chao indices (Figures 3A–D) and 3F–I). However, the coverage index showed extremely significant differences between any two groups (*p* < 0.01) (Figure 3E).

**Figure 4:**
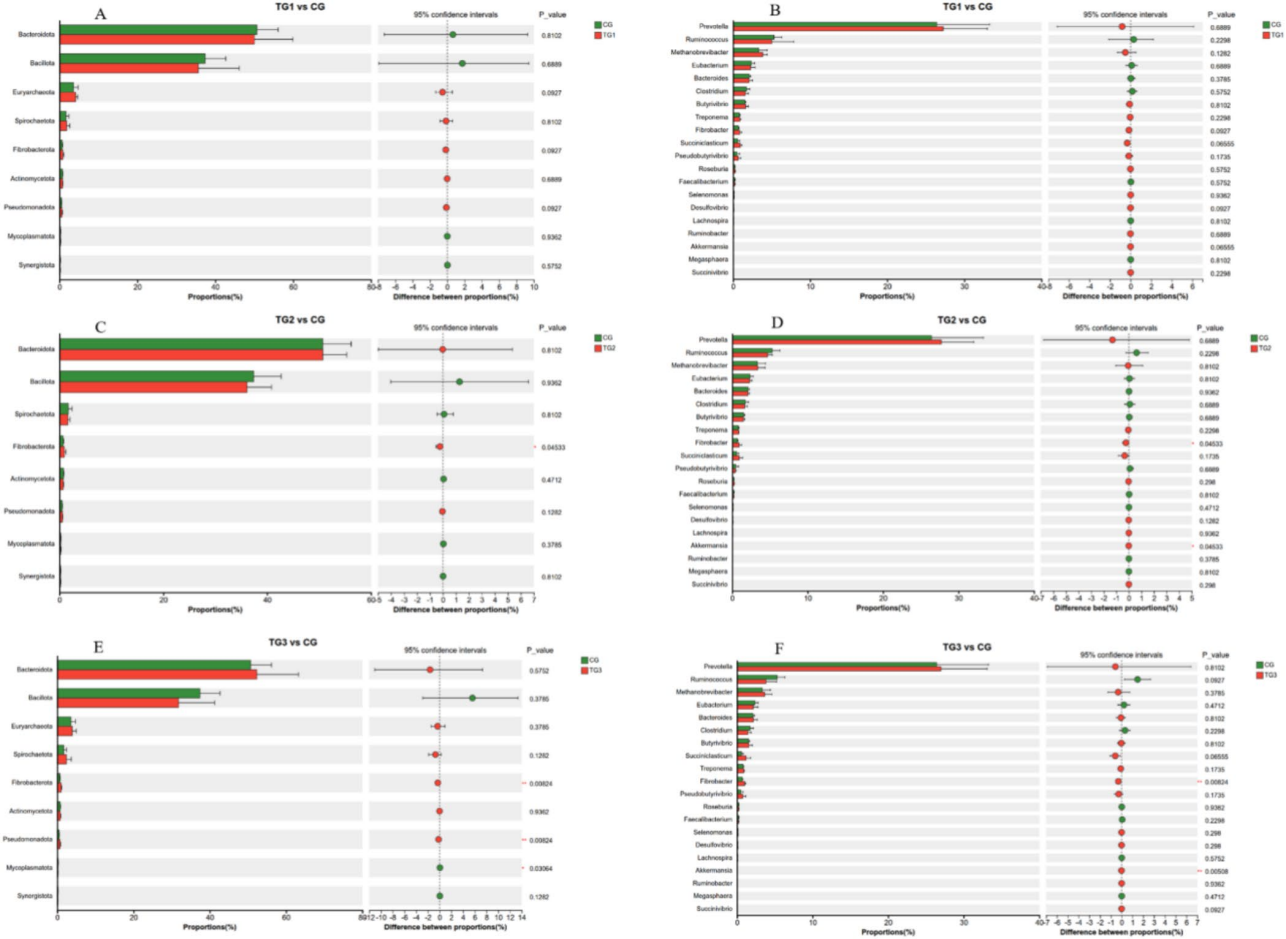
Species with Discrepancies Between Phylum and Genus LevelsBar charts showing differentially abundant taxa for each group: **(A, B)** TG1 vs CG (Phylum,Genus); **(C, D)** TG2 vs CG (Phylum, Genus); **(E, F)** TG3 vs CG (Phylum, Genus).

There was no significant difference in overall rumen microbial community *β*-diversity between the experimental group and the CG. At the phylum level, the relative abundances of phyla such as *Actinobacteria, Bacteroidetes, Proteobacteria,* and *Archaea* associated with energy metabolism and carbon-nitrogen metabolism showed no significant changes (Figures 4A,B). At the genus level, genera including *Prevotella, Fibrobacter,* and *Vibrio butyricus* linked to nitrogen metabolism, acid production through fiber degradation, and fermentation exhibited no significant alterations. Differential abundance analysis revealed that compared with the CG, the abundance of *Fibrobacterota, Fibrobacter,* and *Akkermansia* in the TG2 (Figures 4C,D), as well as *Fibrobacter* and *Akkermansia* in the TG3, was significantly increased (*p* < 0.05) (Figures 4E,F).

**Figure 5:**
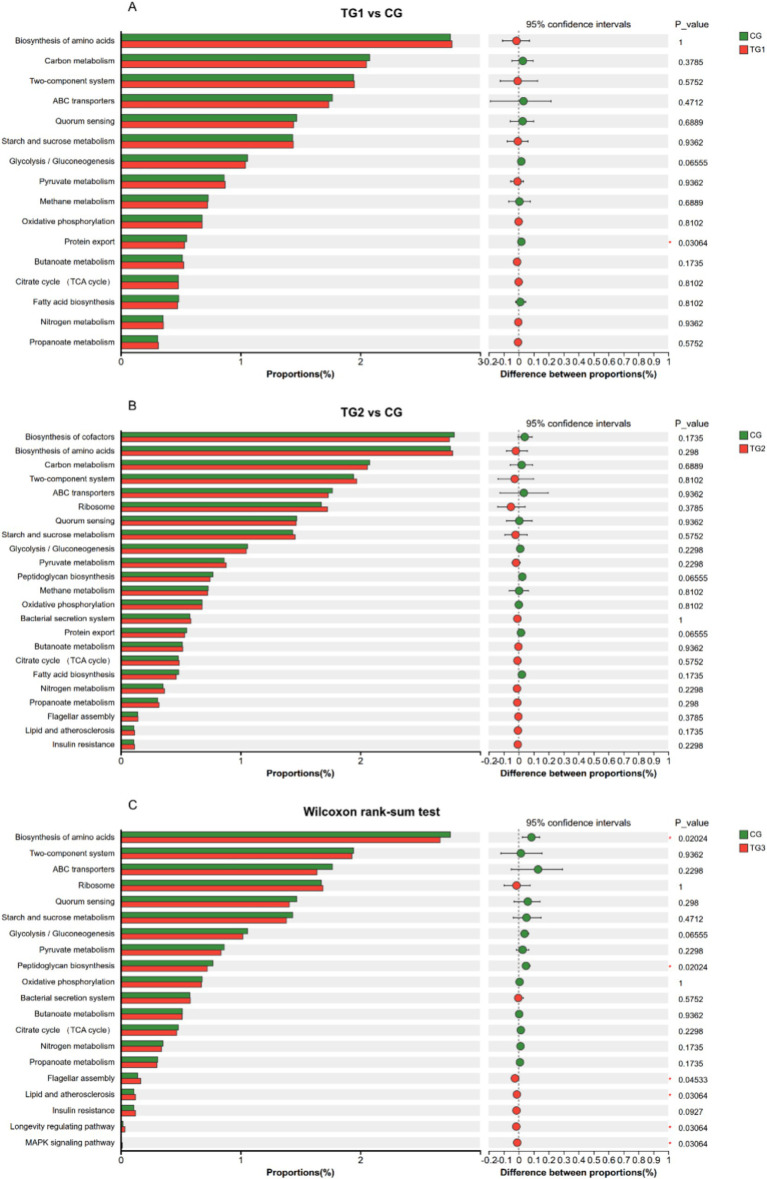
Differential pathway enrichment analysis. **(A)** TG1 vs CG; **(B)** TG2 vs CG; **(C)** TG3 vs CG.

Compared with the CG at KEGG level 3, the TG1 showed significantly upregulated enrichment in pathways such as Longevity regulating pathway-worm and Tuberculosis, while pathways like Protein export were significantly downregulated (*p* < 0.05) (Figure 5A). The TG2 exhibited significant upregulation in pathways including Flavone and flavonol biosynthesis, and significant downregulation in pathways such as Nucleotide metabolism (*p* < 0.05) (Figure 5B). In the TG3, the Longevity regulating pathway and MAPK signaling pathway were significantly up-regulated, while Biosynthesis of amino acids, Peptidoglycan biosynthesis, Biosynthesis of secondary metabolites, and Biosynthesis of amino acids were significantly down-regulated (*p* < 0.05) (Figure 5C).

**Figure 6:**
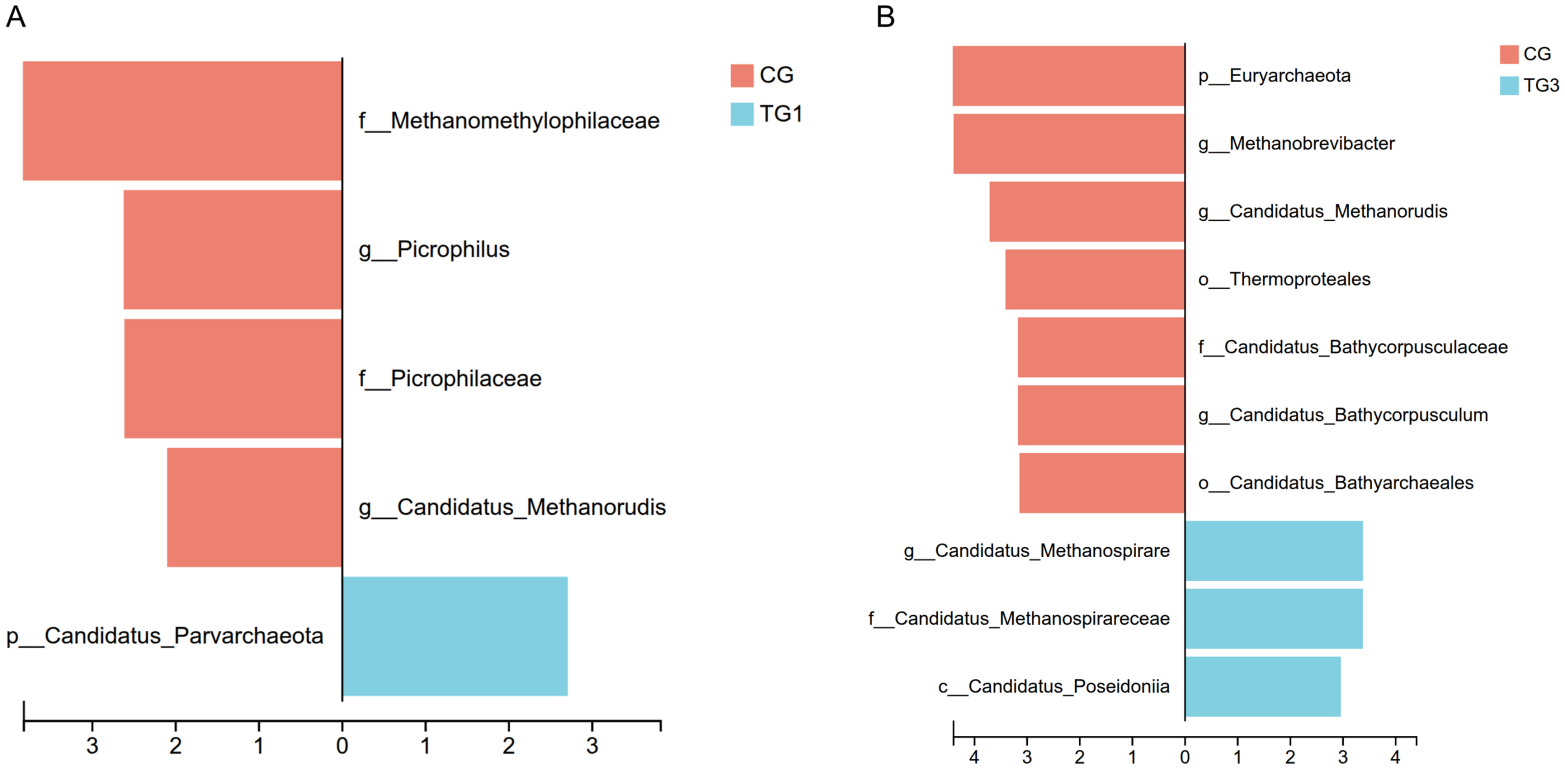
LEfSe analysis of species with intergroup differences. **(A)** TG1 vs CG; **(B)** TG3 vs CG.

Analysis of intergroup differences among the top 10 species with LDA > 2 revealed that in the TG1 vs. CG analysis, the core dominant microorganisms in the CG were *Methanomethylophilaceae* and *Candidatus Methanorudis*, while the TG1 showed significant enrichment of the marker group *Ca. Parvarchaeota* (Figure 6A). In the TG2 vs. CG analysis, no unique biomarkers were identified. In the TG3 vs. CG analysis, the CG exhibited microbial signatures including *Euryarchaeota, Methanobrevibacter,* and *Ca. Methanorudis,* while the TG3 included *Ca. Methanospirare* and *Ca. Methanospirareceae.* Although the specific methanogenic archaeal taxa changed, the community remained dominated by methanogens (Figure 6B).

**Figure 7:**
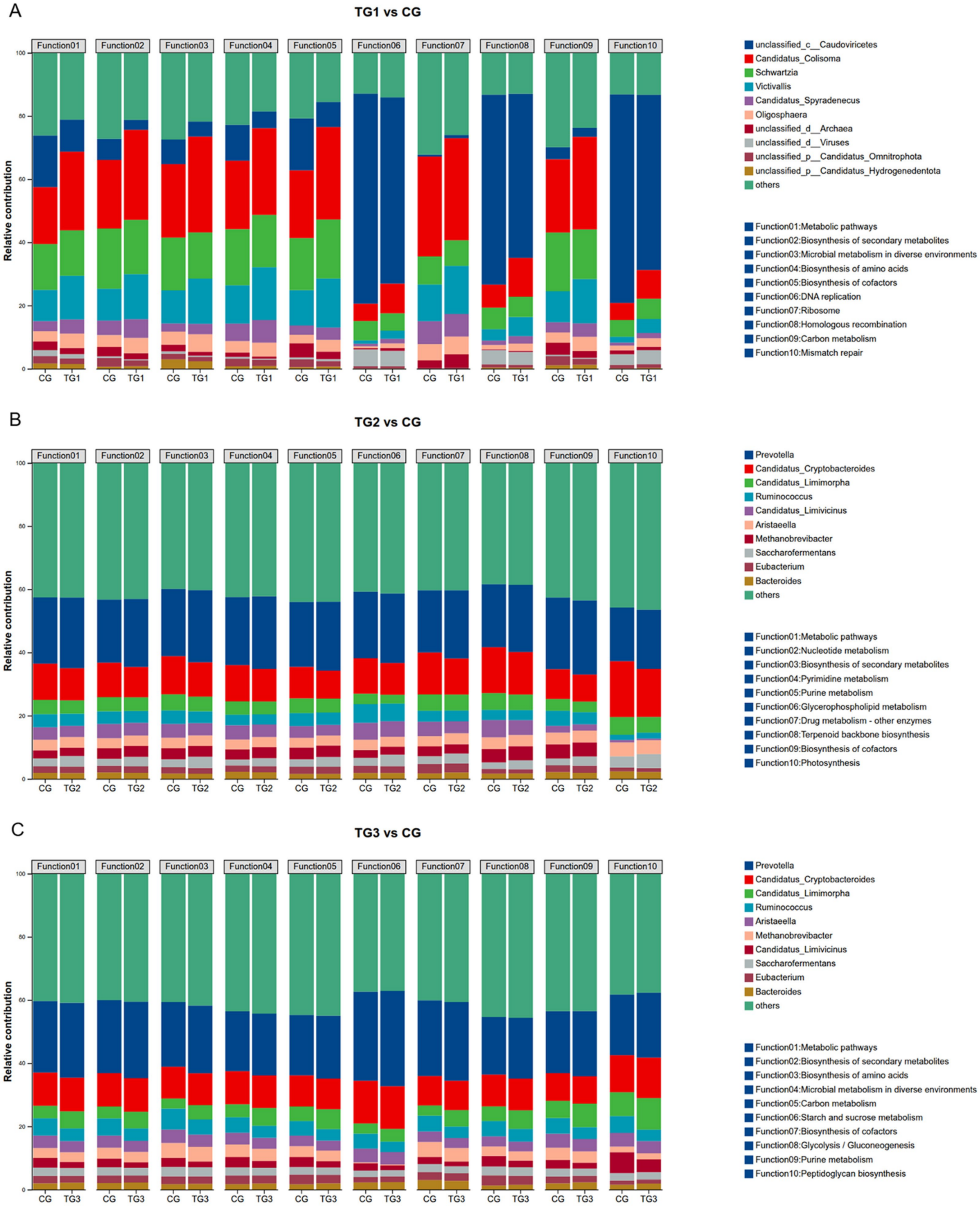
Analysis of species and functional contributions with intergroup differences. **(A)** TG1 vs CG; **(B)** TG2 vs CG; **(C)** TG3 vs CG.

Compared to the CG, the *Caudoviricetes* and *Ca. Colisoma* species in TG1 contributed the largest functional share, collectively accounting for over 70% and jointly dominating functional contributions. This pattern exhibited a dual-species dominance with minor supplementation from other species (Figure 7A). In TG2 and TG3, *Prevotella* dominated functional species contributions. Within the TG2, the functions “Biosynthesis of secondary metabolites and “Starch and sucrose metabolism” exhibited a *Prevotella*-dominated pattern supplemented by multiple other species. The functional contribution species composition remained relatively stable across both groups. In the TG3, species such as *Ca. Cryptobacterium, Ca. Liminiphila*, and Bifidobacterium showed relatively lower contributions (Figures 7B,C).

### 3.2 Nutrient utilization

**Figure 8:**
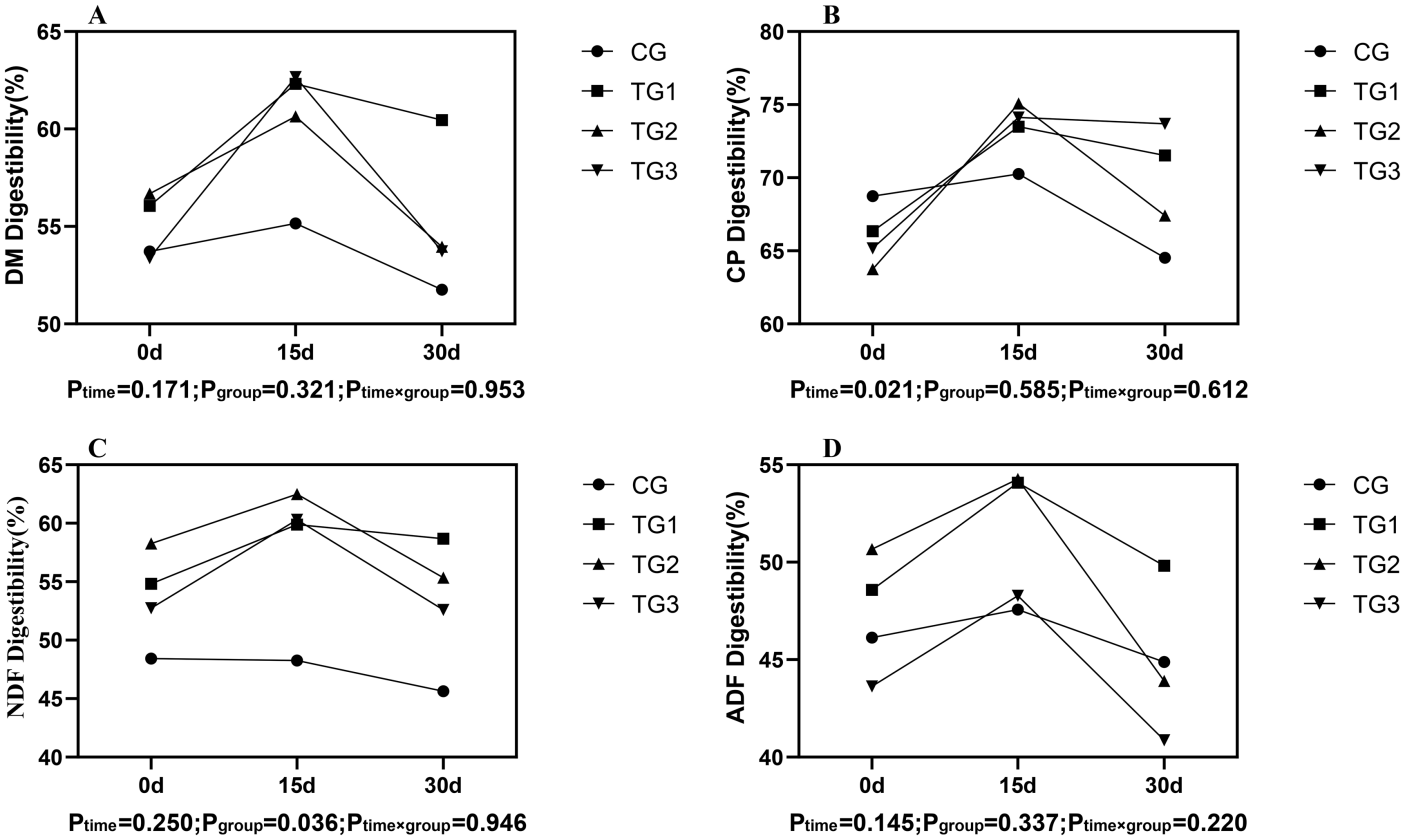
Apparent digestibility of **(A)** DM, **(B)** CP, **(C)** NDF, and **(D)** ADF.

As shown in Figure 8, CPD exhibited significant variation under the main effect of time (*p* < 0.05), while the main effects of grouping for NDFD and ADFD were not significant. Different protein sources did not significantly affect the apparent digestibility of cows within the first month postpartum.

**Figure 9:**
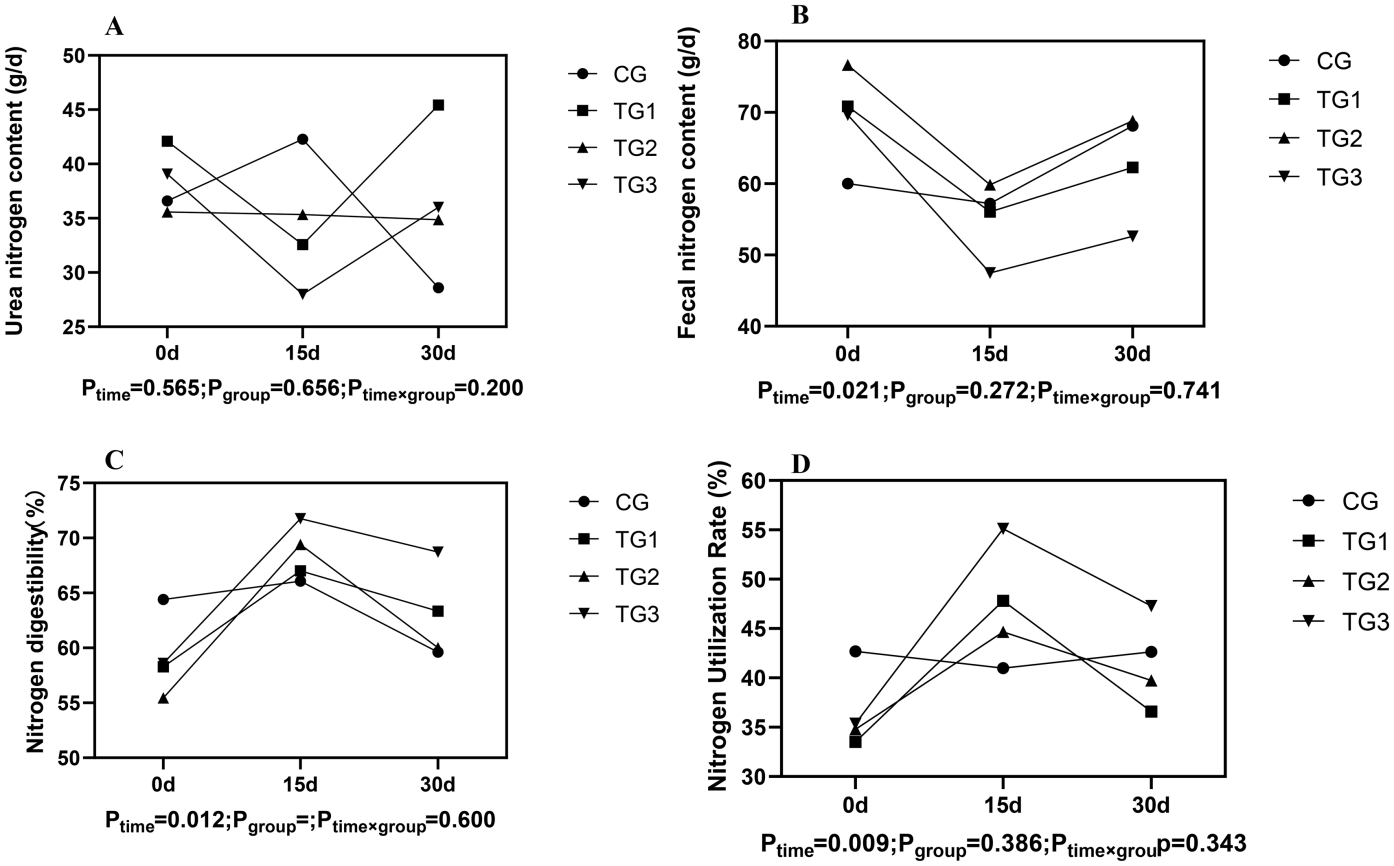
Nitrogen metabolism indicators. **(A)** Urea nitrogen content; **(B)** Fecal nitrogen content; **(C)** Nitrogen utilizatibility; **(D)** Nitrogen digestion rate.

As shown in Figure 9, no significant changes were observed in fecal nitrogen, urinary nitrogen, nitrogen digestibility, or nitrogen utilization rate across different time periods among the groups.

**Figure 10:**
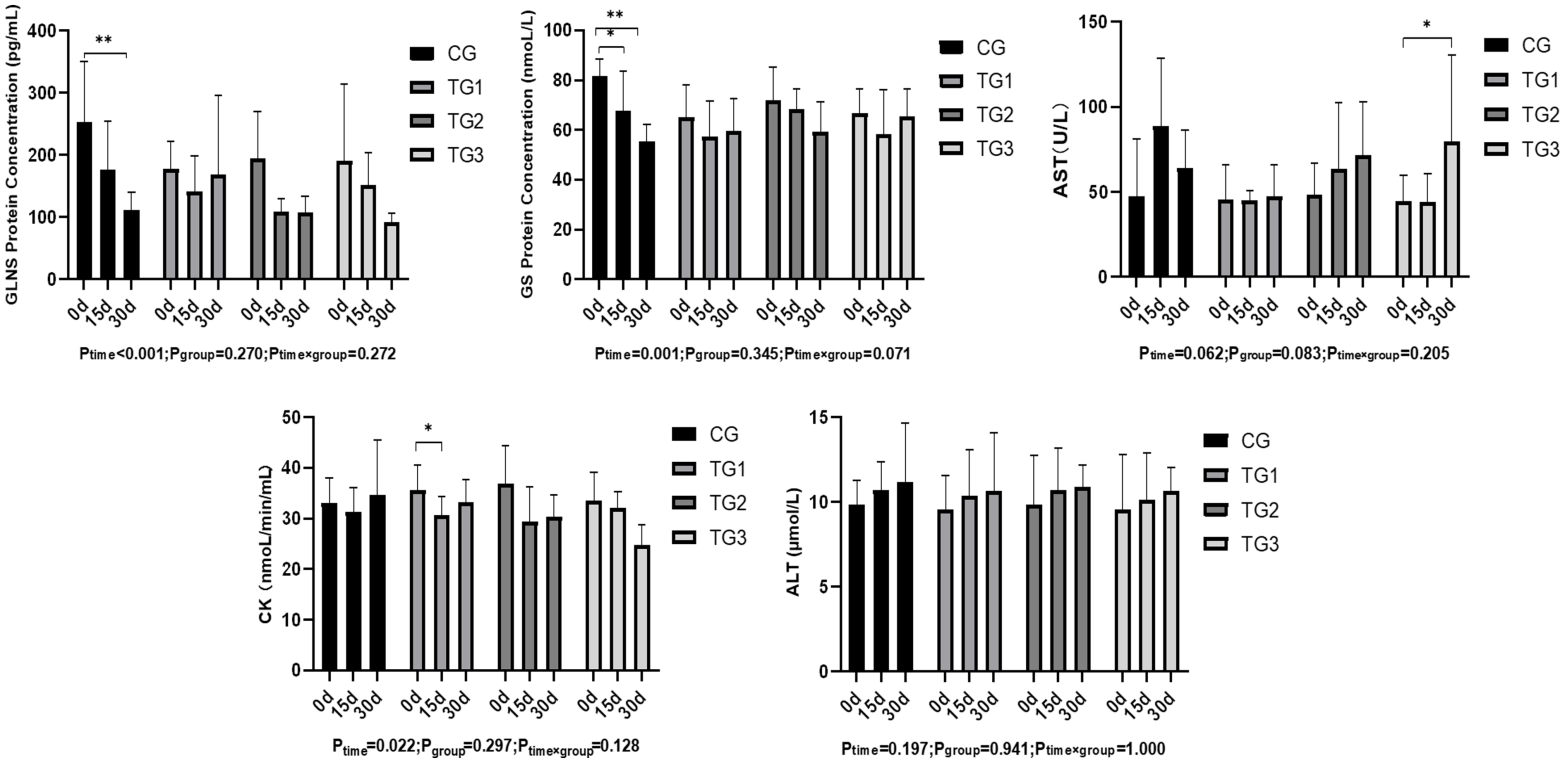
Key enzymes in serum protein metabolism.

As shown in Figure 10, GLNS and GS in the CG at 0 d were significantly higher than PP30 d (*p* < 0.01). AST in the TG3 at PP30 d was significantly higher than 0 d (*p* < 0.05). CK in the TG2 and GS in the CG at 0 d were significantly higher than PP15 d (*p* < 0.05). The time and grouping interactions were not significant across all groups.

**Figure 11:**
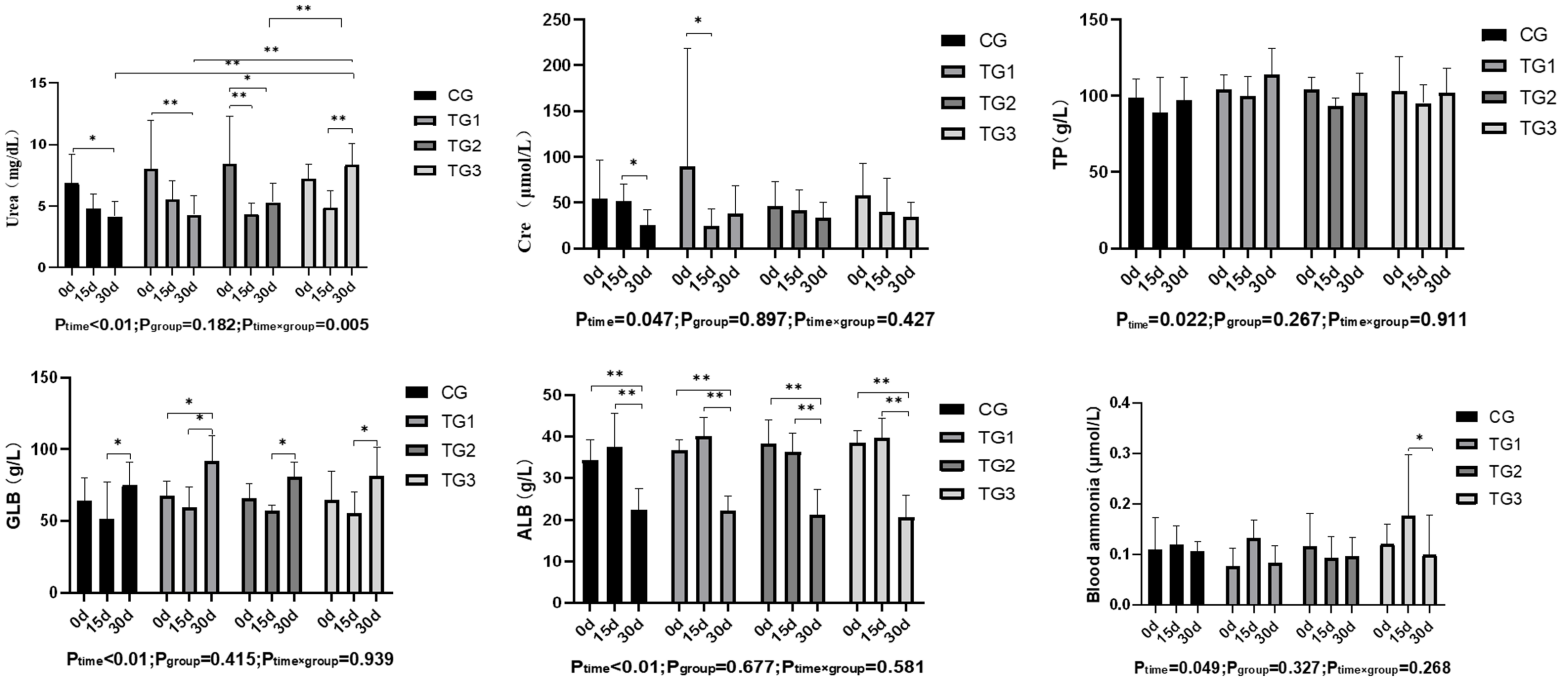
Nitrogen metabolism indicators.

As shown in Figure 11, the TG3 exhibited significantly elevated urea levels at PP30 d, contrasting with the significant downward trends observed in the CG and TG1 at PP30 d and the TG2 at PP15 d. Furthermore, the TG3 urea levels at PP30 d were significantly higher than those in all other groups (*p* < 0.05). Cre was significantly reduced in the CG at PP30 d and in the TG1 at PP15 d. Blood ammonia was significantly reduced in the TG3 at PP30 d. All groups exhibited significantly reduced ALB and significantly increased GLB at PP30 d (*p* < 0.05).

**Figure 12:**
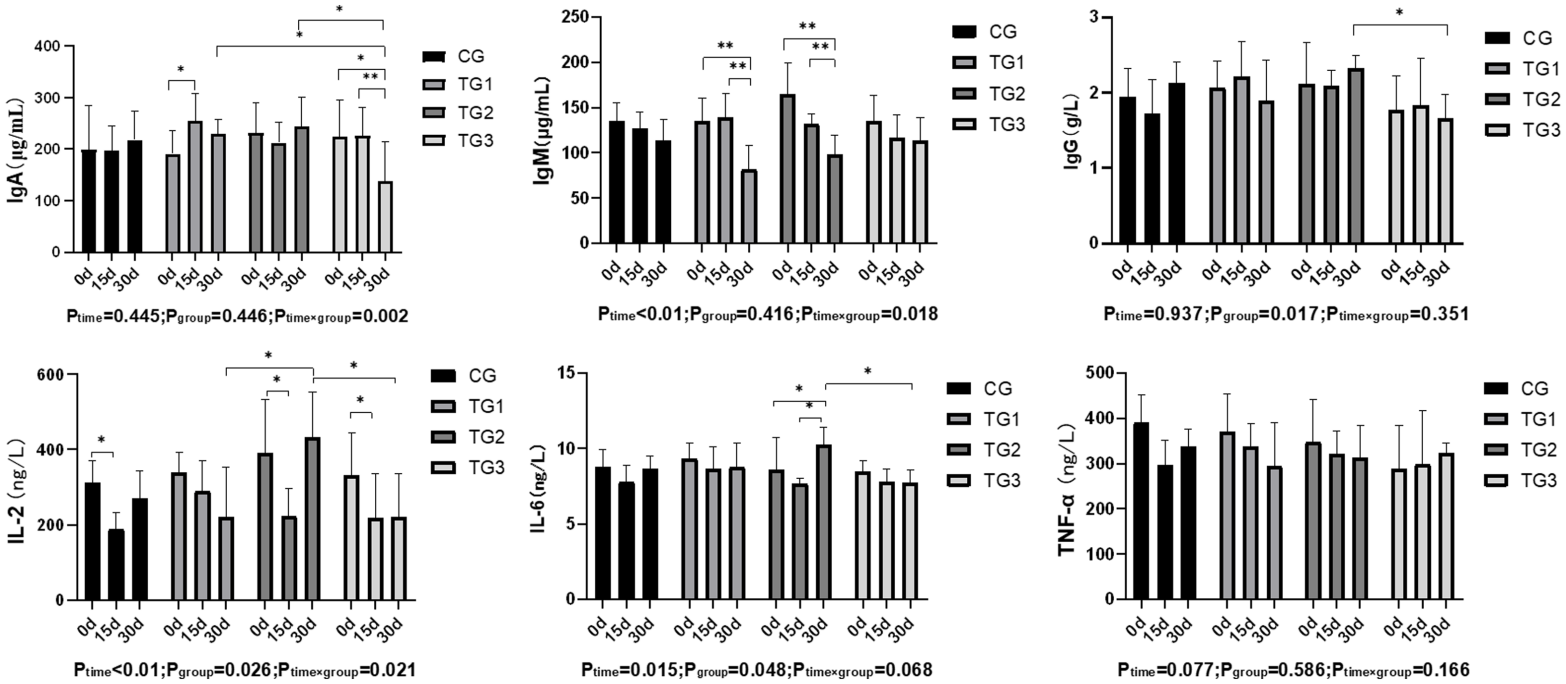
Immunological and inflammatory markers.

Serum IgM levels at 0 d were significantly higher in TG1 and TG2 compared to PP15 d and PP30 d (*p* < 0.01). IgA at PP30 d was significantly higher in TG1 and TG2 than in TG3; TG1 showed significantly higher levels at PP15 d than PP30 d; TG3 exhibited significantly higher levels at 0 d than PP15 d and PP30 d (*p* < 0.05). TG2 30 d IgG was significantly higher than TG3 (*p* < 0.05); CG, TG2, and TG3 0 d IL-2 was significantly higher than PP15 d, and TG2 at PP30 d was significantly higher than TG1 and TG3 (*p* < 0.05); TG2 showed significantly higher IL-6 levels at PP30 d compared to both 0 d and PP15 d, and significantly higher than TG3 PP30 d (*p* < 0.05). IgA, IgM, and IL-2 levels exhibited significant interactions between time and group factors (*p* < 0.05) (Figure 12).

**Figure 13:**
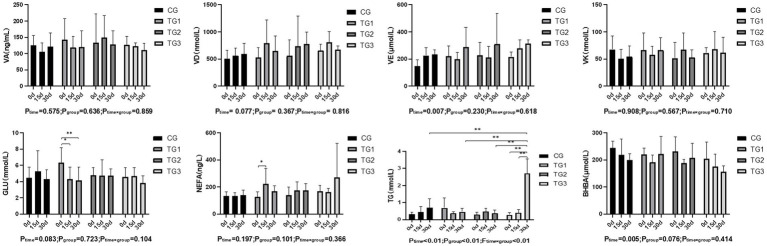
Biochemical indicators.

As shown in Figure 13, Glu levels in the TG1 at 0 d were extremely significantly higher than PP30 d (*p* < 0.01) and significantly higher than PP15 d (*p* < 0.05). NEFA levels at 0 d were significantly lower than PP15 d (*p* < 0.05). Serum TG levels at PP30 d in the TG3 were extremely significantly higher than in other groups (*p* < 0.01). No significant changes were observed in VA, BHBA, or other parameters.

**Figure 14:**
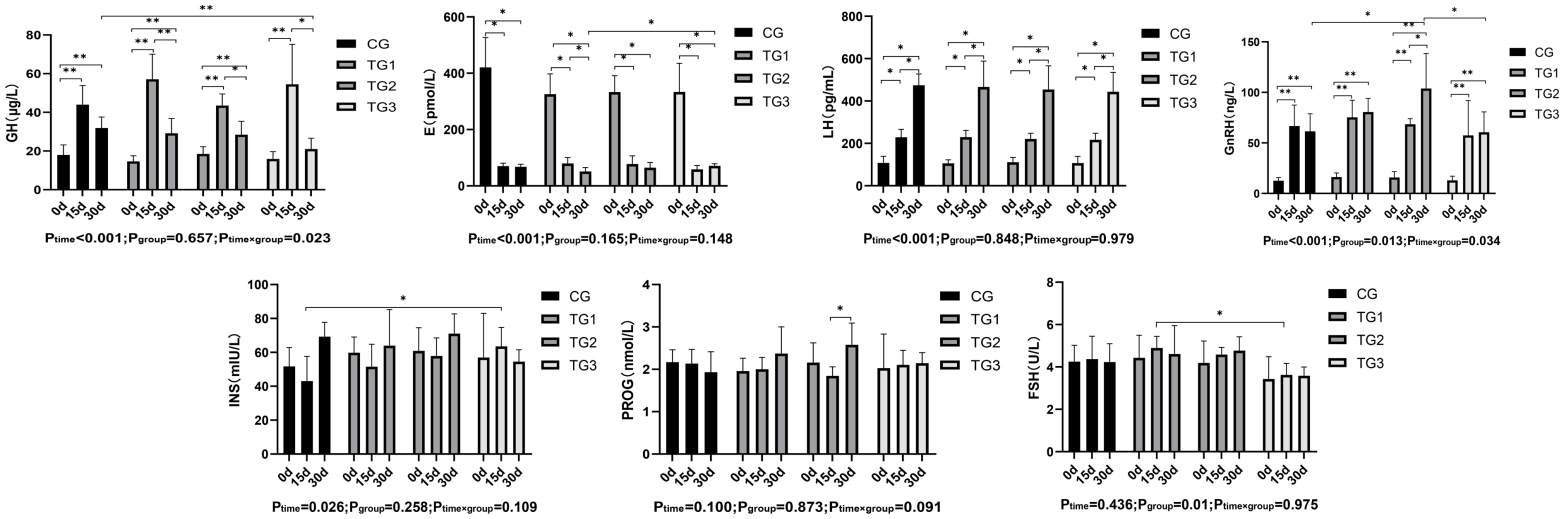
Reproduction-related hormones.

As shown in Figure 14, GH levels in serum at 0 d were significantly elevated across all groups (*p* < 0.01), followed by a marked decline after peaking at PP15 d. Concurrently, TG3 exhibited significantly lower levels at PP30 d compared to CG (*p* < 0.05). TG3 showed significantly higher INS at PP15 d compared to CG (*p* < 0.05); LH and GnRH levels in all groups began to rise significantly at 0 d (*p* < 0.05). GnRH in TG2 was significantly higher than CG and TG3 at PP30 d, and extremely significantly higher than 0 d and PP15 d (*p* < 0.01); Serum E levels were significantly reduced at 0 d in all groups, tending toward equilibrium between PP15 d and PP30 d (*p* < 0.05); PROG levels at PP30 d were significantly higher in the TG2 than at PP15 d, while FSH levels at PP15 d were significantly lower in the TG3 than in the TG1 (*p* < 0.05).

As shown in Tables 6, 7, TG2 exhibited significantly higher levels of Met, Tyr, and His at 0 d compared to TG1. TG1 demonstrated significantly higher Leu levels at PP15 d than CG, while TG2 showed significantly lower Tyr levels at PP30 d than CG (*p* < 0.05). In the TG1, Val, Ile, Phe, Tyr, and His at 0 d were significantly lower than PP15 d and PP30 d, while Met at PP30 d was significantly higher than at 0 d. In both the CG and TG3, Tyr at PP15 d and PP30 d was significantly higher than at 0 d. Additionally, PP30 d Phe in the TG3 was significantly higher than at 0 d, and Phe at PP30 d in the CG was significantly higher than both 0 d and PP15 d (*p* < 0.05).

**Table 6:** Rumen microbial synthesis-dominated AA.

Item	Days postpartum	CG	TG1	TG2	TG3	F	*p*
Val (mg/dL)	0 d	2.52 ± 0.65	2.42 ± 0.38	2.92 ± 0.42	2.58 ± 0.52	0.288	0.128
	15 d	2.74 ± 0.63	3.36 ± 0.58^a^	3.01 ± 0.40	2.80 ± 0.23	0.072	0.225
	30 d	3.02 ± 0.62	3.12 ± 0.65^a^	2.79 ± 0.70	2.79 ± 0.38	0.610	0.064
	F	1.933	9.310	0.317	0.594		
	*p*	0.165	0.001	0.731	0.559		
Met (mg/dL)	0 d	0.38 ± 0.10	0.32 ± 0.05	0.46 ± 0.08^B^	0.36 ± 0.05	4.394	0.012
	15 d	0.36 ± 0.11	0.39 ± 0.07	0.43 ± 0.12	0.33 ± 0.07	1.499	0.237
	30 d	0.40 ± 0.07	0.44 ± 0.09^a^	0.38 ± 0.08	0.35 ± 0.08	1.756	0.179
	F	0.465	3.449	1.509	0.214		
	*p*	0.633	0.047	0.240	0.809		
ILe (mg/dL)	0 d	1.32 ± 0.35	1.20 ± 0.23	1.47 ± 0.24	1.47 ± 0.44	0.308	0.123
	15 d	1.53 ± 0.30	1.83 ± 0.30^a^	1.56 ± 0.24	1.57 ± 0.24	0.132	0.185
	30 d	1.64 ± 0.31	1.61 ± 0.30^a^	1.41 ± 0.31	1.55 ± 0.19	0.446	0.092
	F	3.117	11.713	0.504	0.316		
	*p*	0.061	<0.001	0.610	0.732		
Leu (mg/dL)	0 d	1.89 ± 0.67	1.91 ± 0.24	2.31 ± 0.33	2.14 ± 0.63	1.147	0.348
	15 d	1.91 ± 0.30	2.37 ± 0.30^A^	2.14 ± 0.23	2.05 ± 0.31	3.547	0.028
	30 d	2.09 ± 0.44	2.16 ± 0.43	1.99 ± 0.31	2.01 ± 0.29	0.329	0.804
	F	1.042	2.847	1.513	0.274		
	*p*	0.367	0.076	0.239	0.763		
Tyr (mg/dL)	0 d	0.84 ± 0.17	0.67 ± 0.16	0.90 ± 0.13^B^	0.67 ± 0.08	5.369	0.005
	15 d	1.05 ± 0.22^a^	1.14 ± 0.18^a^	1.11 ± 0.14	1.03 ± 0.09^a^	0.787	0.512
	30 d	1.18 ± 0.28^a^	1.11 ± 0.23^a^	0.88 ± 0.14^A^	0.92 ± 0.11^a^	4.154	0.015
	F	9.386	23.878	3.325	11.095		
	*p*	0.001	<0.001	0.052	<0.001		
Phe (mg/dL)	0 d	0.97 ± 0.27	0.91 ± 0.18	1.20 ± 0.27	0.95 ± 0.15	2.638	0.070
	15 d	0.93 ± 0.23	1.16 ± 0.10^a^	1.03 ± 0.18	1.05 ± 0.17	2.429	0.087
	30 d	1.33 ± 0.20^ab^	1.33 ± 0.28^a^	1.21 ± 0.15	1.18 ± 0.12^a^	1.274	0.303
	F	10.791	11.706	1.744	3.485		
	*p*	<0.001	<0.001	0.195	0.046		
His (mg/dL)	0 d	1.14 ± 0.30	1.02 ± 0.11	1.44 ± 0.24^B^	1.21 ± 0.15	5.190	0.006
	15 d	1.17 ± 0.28	1.31 ± 0.12^a^	1.25 ± 0.12	1.23 ± 0.22	0.689	0.567
	30 d	1.37 ± 0.27	1.37 ± 0.31^a^	1.27 ± 0.19	1.33 ± 0.19	0.285	0.835
	F	2.696	5.577	1.651	0.658		
	*p*	0.086	0.010	0.211	0.526		

Note: a indicates comparison with 0 d, *p* < 0.05 b indicates comparison with 15 d, *p* < 0.05; A indicates comparison with CG, *p* < 0.05; B indicates comparison with TG1, *p* < 0.05; C indicates comparison with TG2, *p* < 0.05.

**Table 7:** Overall evaluation of AA dominated by rumen microbial synthesis.

Item	F_group_	F_Time_	F_Time × group_	P_group_	P_Time_	P_Time × group_
Val	0.833	5.270	1.837	0.488	0.008	0.109
Met	4.091	0.222	1.718	0.016	0.801	0.134
ILe	0.170	7.800	2.454	0.916	0.001	0.036
Leu	0.707	0.296	1.966	0.556	0.746	0.087
Tyr	3.911	27.532	3.155	0.019	<0.001	0.012
Phe	0.488	16.757	3.012	0.488	<0.001	0.013
His	0.647	3.959	2.832	0.597	0.034	0.018

As shown in Tables 8, 9, the CG exhibited significantly higher Thr content at 0 d compared to the experimental group, and significantly higher at PP15 d than the TG3. The TG1 and TG2 showed significantly lower Thr content at 0 d than PP15 d and PP30 d, while the TG3 demonstrated significantly higher Thr content at PP15 d than 0 d (*p* < 0.05). The TG2 exhibited significantly lower Cys levels at PP30 d compared to PP15 d (*p* < 0.05). The main effects of time on both Thr and Cys were highly significant (*p* < 0.01).

**Table 8:** Rumen-bypass AA in diets.

Iterm	Days postpartum	CG	TG1	TG2	TG3	*F*	*p*
Thr (mg/dL)	0 d	4.03 ± 1.35	0.91 ± 0.13^A^	1.08 ± 0.16^A^	1.13 ± 0.18^A^	36.238	<0.001
	15 d	4.55 ± 0.85	4.4 ± 0.54^a^	4.05 ± 0.84^a^	3.33 ± 0.98^Aa^	3.563	0.027
	30 d	5.31 ± 0.78	5.76 ± 1.34^a^	7.39 ± 10.45^a^	3.44 ± 0.28	0.799	0.505
	F	1.032	40.736	27.968	15.704		
	*p*	0.371	<0.001	<0.001	<0.001		
Cys (mg/dL)	0 d	0.19 ± 0.13	0.14 ± 0.04	0.17 ± 0.04	0.19 ± 0.13	0.460	0.713
	15 d	0.21 ± 0.03	0.23 ± 0.03	0.23 ± 0.04	0.23 ± 0.05	0.409	0.748
	30 d	0.16 ± 0.06	0.22 ± 0.09	0.13 ± 0.06^b^	0.16 ± 0.06	2.520	0.079
	F	1.486	2.718	4.574	2.500		
	*p*	0.245	0.085	0.020	0.102		
Lys (mg/dL)	0 d	1.23 ± 0.44	1.03 ± 0.10	1.45 ± 0.21	1.31 ± 0.48	1.930	0.149
	15 d	1.33 ± 0.36	1.35 ± 0.18	1.36 ± 0.17	1.33 ± 0.25	0.030	0.993
	30 d	1.52 ± 0.43	1.39 ± 0.32	1.38 ± 0.24	1.36 ± 0.26	0.401	0.753
	F	1.605	4.062	0.228	0.038		
	*p*	0.220	0.029	0.797	0.963		
Arg (mg/dL)	0 d	0.89 ± 0.24	1.33 ± 0.93	1.19 ± 0.24	0.91 ± 0.15	1.439	0.253
	15 d	0.89 ± 0.42	1.09 ± 0.26	0.96 ± 0.15	1.00 ± 0.13	0.745	0.535
	30 d	1.27 ± 0.26	1.44 ± 0.73	1.14 ± 0.30	1.04 ± 0.21	1.278	0.302
	F	2.297	2.145	0.803	0.154		
	*p*	0.121	0.137	0.459	0.858		

Note: a indicates comparison with 0 d, *p* < 0.05 b indicates comparison with 15 d, *p* < 0.05; A indicates comparison with CG, *p* < 0.05; B indicates comparison with TG1, *p* < 0.05; C indicates comparison with TG2, *p* < 0.05.

**Table 9:** Overall evaluation of rumen-bypass AA in diets.

Item	F_group_	F_Time_	F_Time × group_	P_group_	P_Time_	P_Time × group_
Thr	2.053	12.193	1.288	0.130	<0.001	0.278
Cys	0.295	5.547	1.486	0.829	0.006	0.201
Lys	0.639	2.266	1.199	0.597	0.114	0.321
Arg	2.424	2.666	0.689	0.087	0.079	0.068

As shown in Tables 10, 11, TG2 exhibited significantly higher Asp levels at 0 d compared to CG and TG1. TG1 demonstrated significantly higher Asp levels at PP15 d than 0 d and PP30 d (*p* < 0.05), while TG2 showed significantly lower Asp levels at PP30 d than 0 d (*p* < 0.05). Ser in the CG at PP30 d was significantly higher than 0 d and PP15 d, while in the TG1, Ser at PP30 d was significantly higher than at 0 d (*p* < 0.05). Glu at 0 d in the TG2 and at PP15 d in the TG1 and TG3 was significantly higher than in the CG (*p* < 0.05). Gly at PP30 d in the TG1 was significantly higher than 0 d (*p* < 0.05). Significant main effects of time were observed for Ser, Asp., and Gly; significant group main effects were observed for Glu and Asp; and a significant interaction effect between group and time was observed for Ser (*p* < 0.05).

**Table 10:** Key liver metabolic AA.

Iterm	Days postpartum	CG	TG1	TG2	TG3	F	*p*
Asp (mg/dL)	0 d	0.19 ± 0.06	0.22 ± 0.03	0.3 ± 0.05^AB^	0.25 ± 0.03	9.058	<0.001
	15 d	0.26 ± 0.04^a^	0.29 ± 0.03^a^	0.29 ± 0.03	0.28 ± 0.03	2.150	0.117
	30 d	0.21 ± 0.04	0.23 ± 0.06^b^	0.23 ± 0.05^a^	0.24 ± 0.04	0.336	0.799
	F	8.637	11.1000	3.775	2.791		
	*p*	0.001	<0.001	0.036	0.080		
Ser (mg/dL)	0 d	1.19 ± 0.35	0.98 ± 0.1	1.28 ± 0.18	1.17 ± 0.2	2.319	0.098
	15 d	1.27 ± 0.29	1.23 ± 0.16	1.45 ± 0.15	1.31 ± 0.28	1.260	0.308
	30 d	1.61 ± 0.37^ab^	1.41 ± 0.39^a^	1.24 ± 0.16	1.35 ± 0.24	1.955	0.145
	F	5.505	5.867	1.603	1.243		
	*p*	0.010	0.008	0.220	0.305		
Glu	0 d	2.60 ± 0.67	3.03 ± 0.35	3.60 ± 0.65^A^	3.24 ± 0.54	4.167	0.015
	15 d	2.64 ± 0.48	3.32 ± 0.58^A^	3.14 ± 0.24	3.44 ± 0.44^A^	4.745	0.009
	30 d	3.15 ± 0.68	3.15 ± 0.84	2.67 ± 0.72	3.34 ± 0.58	1.159	0.344
	F	1.486	0.683	2.884	0.337		
	*p*	0.245	0.514	0.074	0.717		
Gly (mg/dL)	0 d	4.89 ± 1.14	4.66 ± 1.02	5.08 ± 0.75	4.96 ± 0.98	0.249	0.861
	15 d	5.18 ± 1.46	5.26 ± 0.94	5.63 ± 1.22	5.04 ± 0.69	0.381	0.768
	30 d	6.21 ± 1.59	6.64 ± 2.19^a^	4.57 ± 0.95	5.38 ± 1.18	2.548	0.077
	F	2.910	6.792	0.915	0.297		
	*p*	0.072	0.004	0.413	0.746		
Ala (mg/dL)	0 d	5.31 ± 1.83	4.40 ± 0.69	5.79 ± 1.16	5.13 ± 0.95	1.657	0.200
	15 d	4.75 ± 1.26	5.40 ± 1.06	4.78 ± 1.91	5.69 ± 0.97	0.961	0.425
	30 d	5.91 ± 1.49	5.72 ± 0.68	4.76 ± 0.63	5.87 ± 1.23	1.816	0.168
	F	1.940	2.589	1.448	0.811		
	*p*	0.164	0.094	0.253	0.455		

Note: a indicates comparison with 0 d, *p* < 0.05 b indicates comparison with 15 d, *p* < 0.05; A indicates comparison with CG, *p* < 0.05; B indicates comparison with TG1, *p* < 0.05; C indicates comparison with TG2, *p* < 0.05.

**Table 11:** Overall analysis of key liver metabolic AAs.

Iterm	F_group_	F_Time_	F_Time × group_	P_group_	P_Time_	P_Time × group_
Asp	8.839	12.159	2.177	<0.001	<0.001	0.076
Ser	1.208	8.315	2.327	0.326	0.001	0.045
Glu	4.535	0.078	2.193	0.011	0.925	0.058
Gly	0.588	5.011	2.967	0.628	0.039	0.075
Ala	0.817	1.029	1.631	0.496	0.364	0.157

### 3.3 Fecal and urinary differential metabolites

The metabolomics analyses presented in this section (3.3.1 and 3.3.2) were performed exclusively on fecal and urine samples collected on 0 d. These 0 d metabolic profiles were subsequently used for correlation analysis with longitudinal parameters measured on 0 d, PP15 d and PP30 d (see Section 3.4).

#### 3.3.1 Fecal differential metabolites

**Figure 15:**
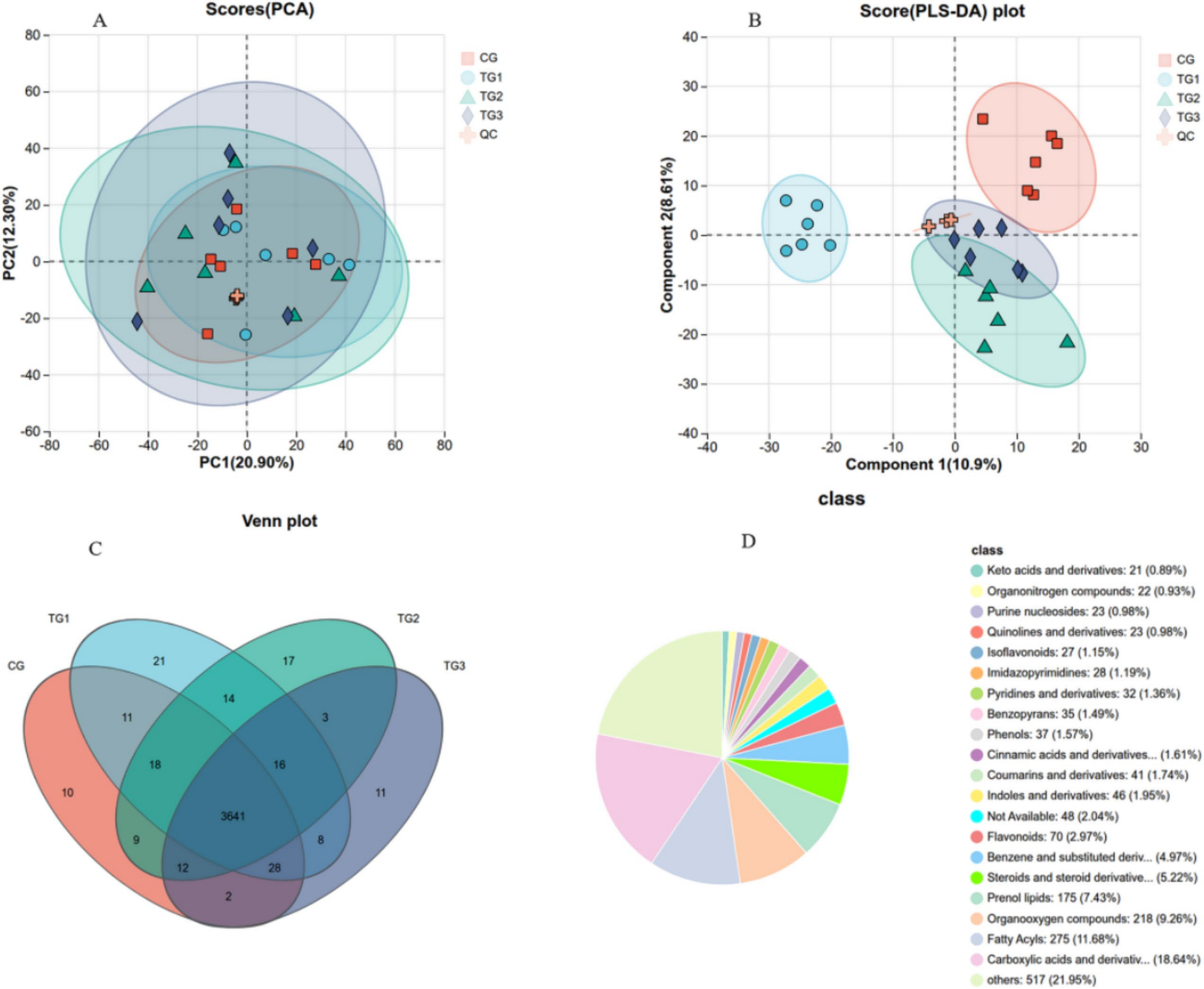
Multivariate analysis and metabolite profiling. **(A)** PCA; **(B)** PCoA; **(C)** Venn analysis; **(D)** HMDB compound classification by class.

As shown in Figure 15A, no significant separation was observed among the groups, with extensive overlap and scattered distribution. PLS-DA analysis indicated that the TG3 and CG exhibited the closest metabolic profiles, suggesting that the TG3 had a relatively minor overall impact on the hindgut metabolism of cows. The TG2 metabolome partially overlaps with that of the TG3. The TG1 metabolome is most distant from the CG and clearly separated from all other groups, indicating that the TG1 exerts the most significant impact on bovine intestinal metabolism (Figure 15B). A total of 3,641 metabolites were shared across all groups, with each group harboring a few unique metabolites: 10 in the CG, 21 in TG1, 17 in TG2, and 11 in TG3 (Figure 15C). HMDB compound classification indicated that fecal metabolites were primarily dominated by three categories: Carboxylic acids and derivatives (18.64%), Fatty Acyls (11.68%), and Organooxygen compounds (9.26%), collectively accounting for approximately 40% of all metabolites. Additionally, Prenol lipids (7.43%), Steroids and steroid derivatives (5.22%), and Benzene and substituted derivatives (4.97%) constituted significant components (Figure 15D).

**Figure 16:**
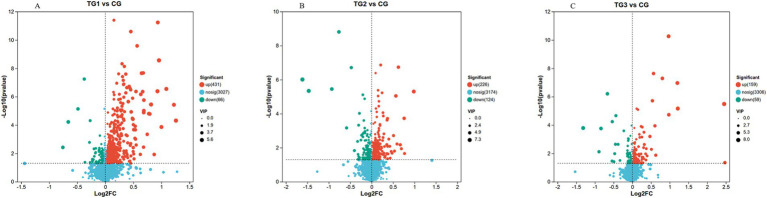
Differentially expressed metabolites. **(A)** TG1 vs CG; **(B)** TG2 vs CG; **(C)** TG3 vs CG.

Using *p*-value<0.05 and VIP > 1 as criteria to screen for differentially expressed metabolites. Compared with the CG, the TG1 showed 497 differentially expressed metabolites (431 up-regulated, 66 down-regulated) (Figure 16A), the TG2 showed 350 (226 up-regulated, 124 down-regulated) (Figure 16B), and the TG3 showed 218 (159 up-regulated, 59 down-regulated) (Figure 16C).

**Figure 17:**
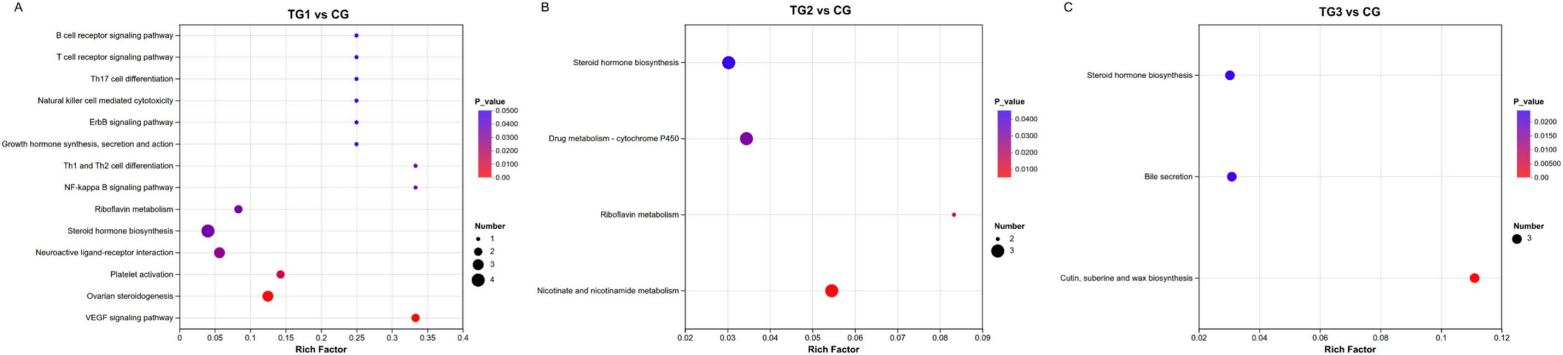
KEGG pathway enrichment analysis. **(A)** TG1 vs CG; **(B)** TG2 vs CG; **(C)** TG3 vs CG.

Differentially expressed metabolites in the TG1 were significantly enriched in steroid hormone biosynthesis, riboflavin metabolism, and various immune and signaling pathways, primarily including steroid hormone biosynthesis, riboflavin metabolism, and the NF-κB signaling pathway (Figure 17A). Differentially expressed metabolites in the TG2 were significantly enriched in pathways such as riboflavin metabolism, steroid hormone biosynthesis, retinol metabolism, and alkaloid biosynthesis (Figure 17B). The TG3 showed significant enrichment in the bile secretion and steroid hormone biosynthesis pathways (Figure 17C).

#### 3.3.2 Urinary differential metabolites

**Figure 18:**
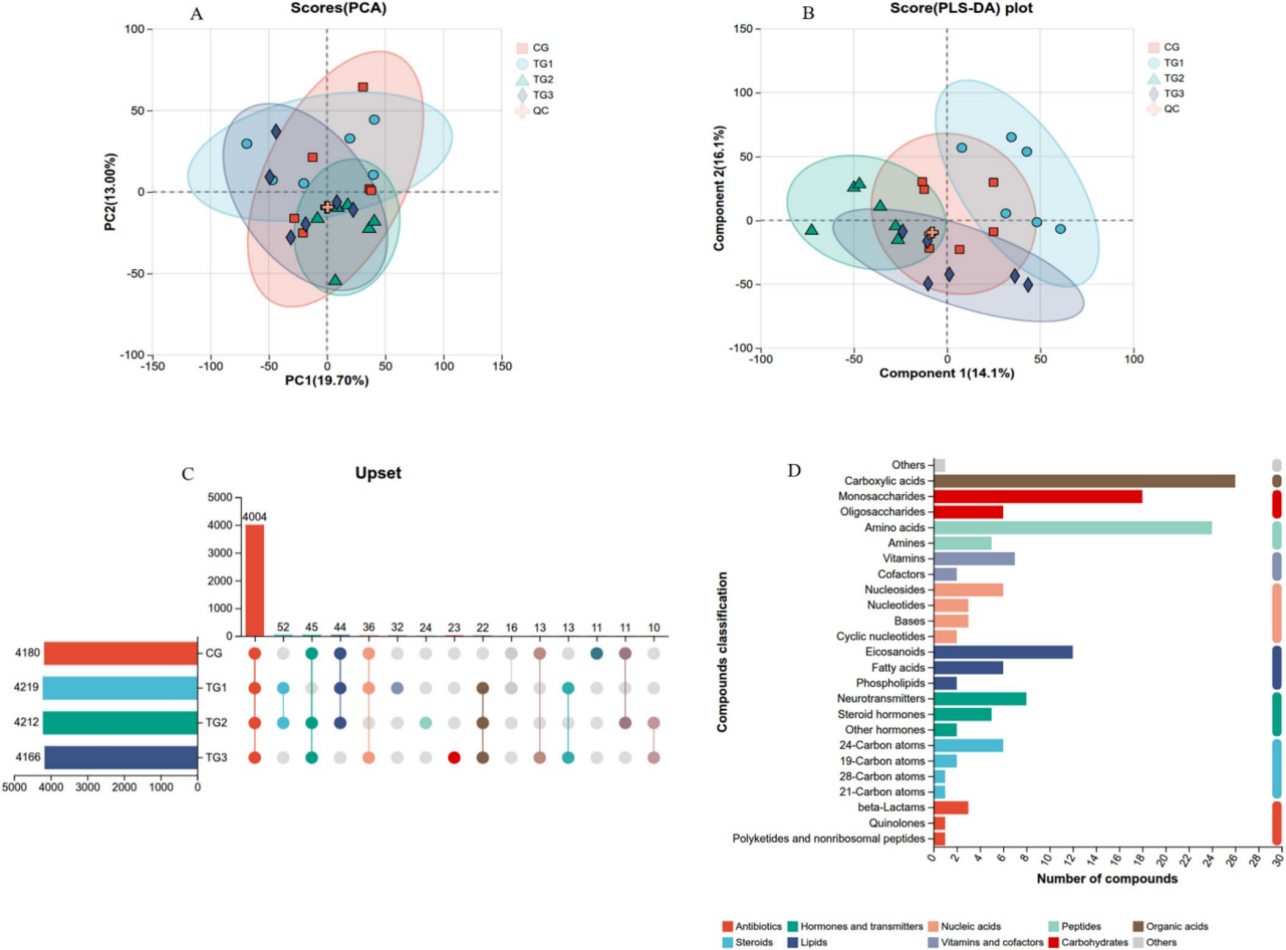
Multimodal analysis of urinary metabolomics data. **(A)** PCA; **(B)** PCoA; **(C)** Venn analysis; **(D)** KEGG compound classification.

PCA and PLS-DA analyses indicate that samples from the experimental groups clustered closely with those from the CG, showing no significant separation (Figures 18A,B). A total of 4,004 metabolites were identified across the urine samples from the four groups. Only a small number of metabolites were unique to each group: 11 in CG, 32 in TG1, 24 in TG2, and 23 in the TG3 (Figure 18C). KEGG compound classification revealed Organic acids as the most abundant group, primarily comprising Carboxylic acids, followed by Peptides encompassing Amino acids and Amines. Other categories included Carbohydrates and Lipids (Figure 18D).

**Figure 19:**
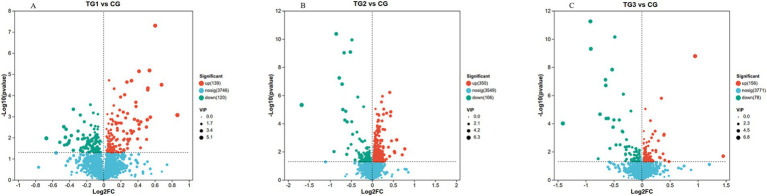
Differentially expressed metabolites. **(A)** TG1 vs CG; **(B)** TG2 vs CG; **(C)** TG3 vs CG.

Differentially expressed metabolites in urine were screened based on *p*-value <0.05 and VIP > 1. Compared with the CG, a total of 259 differentially expressed metabolites were identified in the TG1. Among these, 139 metabolites, including Hypusine, Cysteinyl-Proline, and Isocitric Acid, were upregulated, while 120 metabolites, such as Urea, (S)-Equol, and Equol 7-O-Glucuronide (Figure 19A). The TG2 identified 456 differential metabolites, including 350 upregulated (e.g., Isocitric Acid, Phenylacetic Acid, Propionic Acid) and 106 downregulated (e.g., Urea, 5-Hydroxy-6-Methoxyindole Glucuronide, Morphine-3-Glucuronide) (Figure 19B); The TG3 exhibited 234 differentially expressed metabolites, including 156 upregulated metabolites such as 1-Methylhistidine, Alpha-Linolenic Acid, Homocitrulline, and Trp-Ser-Ala, and 78 downregulated metabolites including Deoxycholic Acid, Cellobiose, and Sphingosine (Figure 19C).

**Figure 20:**
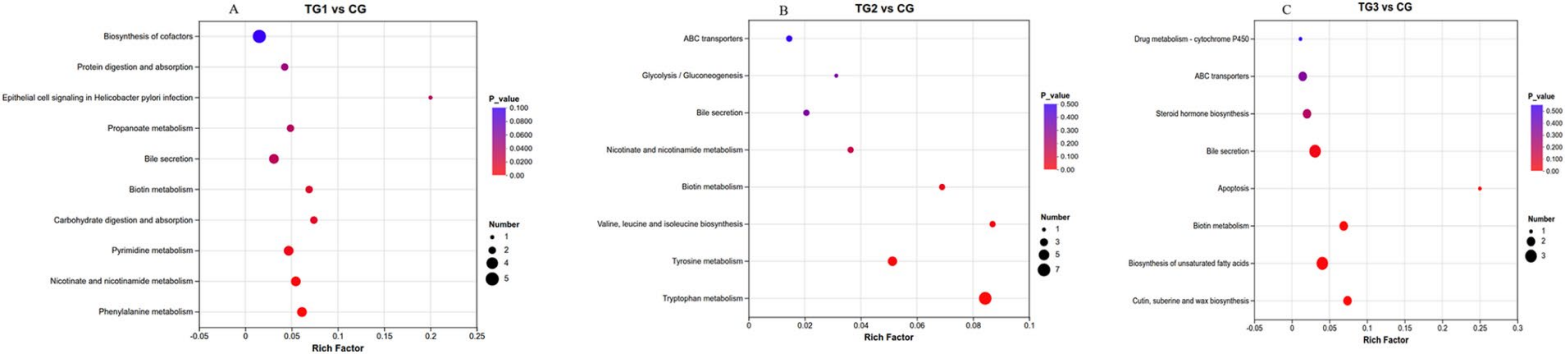
KEGG pathway enrichment analysis. **(A)** TG1 vs CG; **(B)** TG2 vs CG; **(C)** TG3 vs CG.

The TG1 showed significant enrichment in pathways related to protein, carbohydrate digestion and absorption, propanoate metabolism, and amino acid metabolism (*p* < 0.05) (Figure 20A). The TG2 exhibited significant enrichment in tryptophan metabolism, tyrosine metabolism, and branched-chain amino acid biosynthesis pathways (*p* < 0.05) (Figure 20B). The TG3 was primarily enriched in pathways including unsaturated fatty acid biosynthesis, biotin metabolism, and folate biosynthesis (*p* < 0.05) (Figure 20C).

### 3.4 Multi-indicator omics correlation analysis

The correlation analysis in this section examines the relationship between 0 d multi-omics data and physiological parameters measured on 0 d, PP15 d and PP30 d. Correlations with 0 d reflect contemporaneous associations, while correlations with PP15 d and PP30 d reflect cross-temporal associations with subsequent recovery phenotypes.

#### 3.4.1 Correlation analysis of rumen microorganisms and fermentation parameters

**Figure 21:**
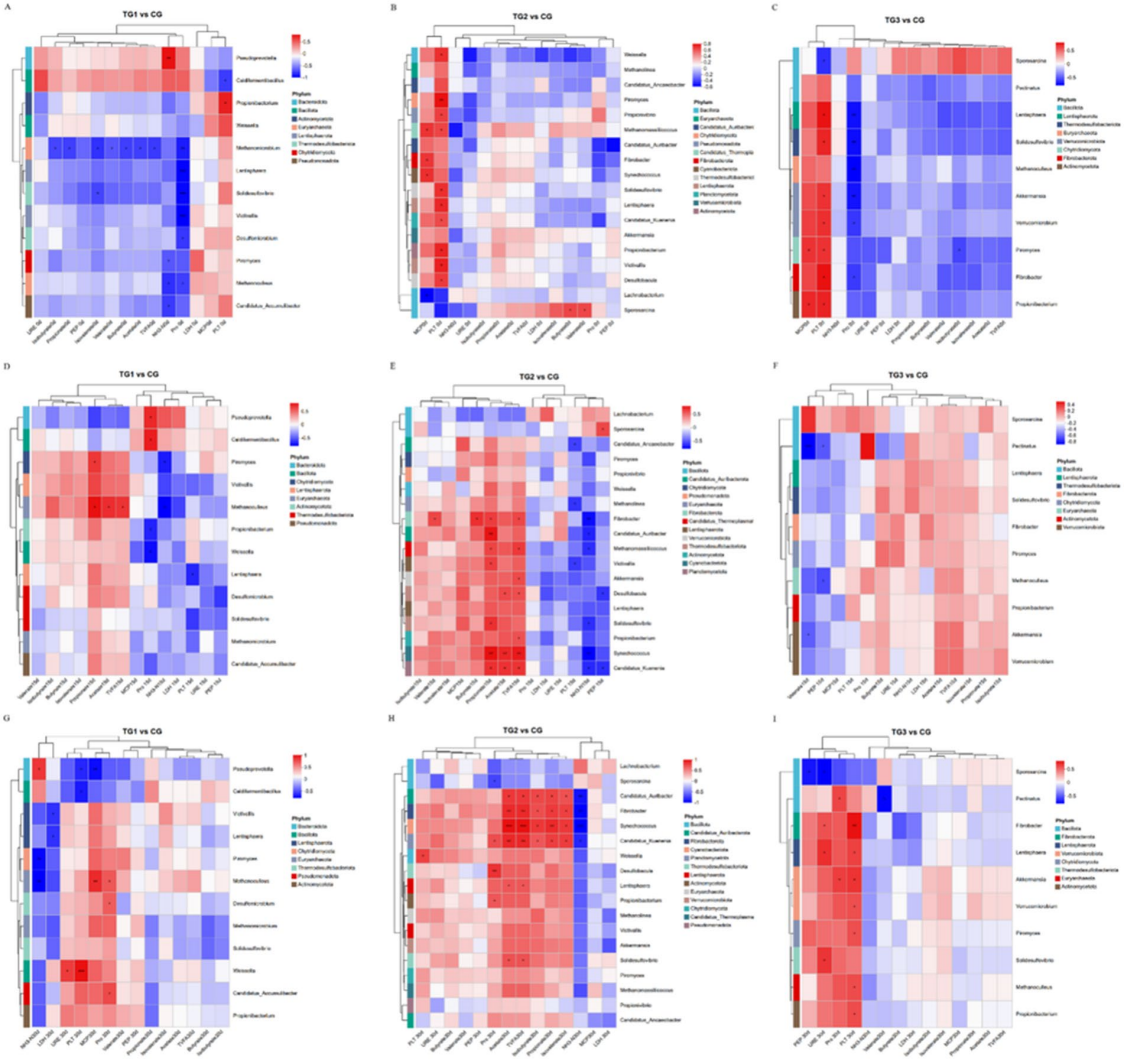
Correlation analysis of fermentation parameters with rumen microbiota at 0 d **(A-C)**, and with fermentation parameters alone at PP15d **(D-F)** and PP 30d **(G-I)** across three treatment groups. TG1: **(A, D, G)**; TG2 **(B, E, H)**; TG3 **(C, F, I)**.Red indicates positive correlation, blue indicates negative correlation; *p < 0.05, **p < 0.01.

At 0 d, the *Pseudoprevotella* of TG1 showed a highly significant or significant positive correlation with NH_3_-N, and *Propionibacterium* showed a highly significant or significant positive correlation with PLT (Figure 21A, *p* < 0.01 or *p* < 0.05). *Fibrobacte*r and *Methanomassiliicoccus* of TG2 were significantly positively correlated with MCP (*p* < 0.05) (Figure 21B, *p* < 0.05). In the TG3, Piromyces, Propionibacterium, and MCP, as well as PLT and *Lentisphaera*, were significantly positively correlated (Figure 21C, *p* < 0.05). At PP15 d, *Methanoculleus* in the TG1 was significantly positively correlated with propionate, acetate, and TVFA (Figure 21D, *p* < 0.05). In the TG2, *Fibrobacter* was significantly positively correlated with propionate, valerate, butyrate, and TVFA (Figure 21E, *p* < 0.05). In The TG3, Valerate showed a highly significant or significant negative correlation with *Pectinatus* and *Akkermansia* (Figure 21F, *p* < 0.01 or < 0.05). At PP30 d, *Methanoculleus* of *T*G1 showed a highly significant or significant positive correlation with MCP and Pro, while *Piromyces* and *Methanoculleus* showed a highly significant or significant negative correlation with NH_3_-N (Figure 21G, *p* < 0.01 or *p* < 0.05). In the TG2, *Fibrobacter, Lentisphaera, Solidesulfovibrio*, and *Synechococcus* showed a highly significant or significant positive correlation with multiple VFA (Figure 21H, *p* < 0.01 or *p* < 0.05). In the TG3, *Fibrobacter* and *Akkermansia* showed significant positive correlations with URE and Pro, while *Pectinatus* showed a significant negative correlation with Valerate (Figure 21I, *p* < 0.05).

#### 3.4.2 Correlation analysis between rumen fermentation parameters and serum indicators

**Figure 22:**
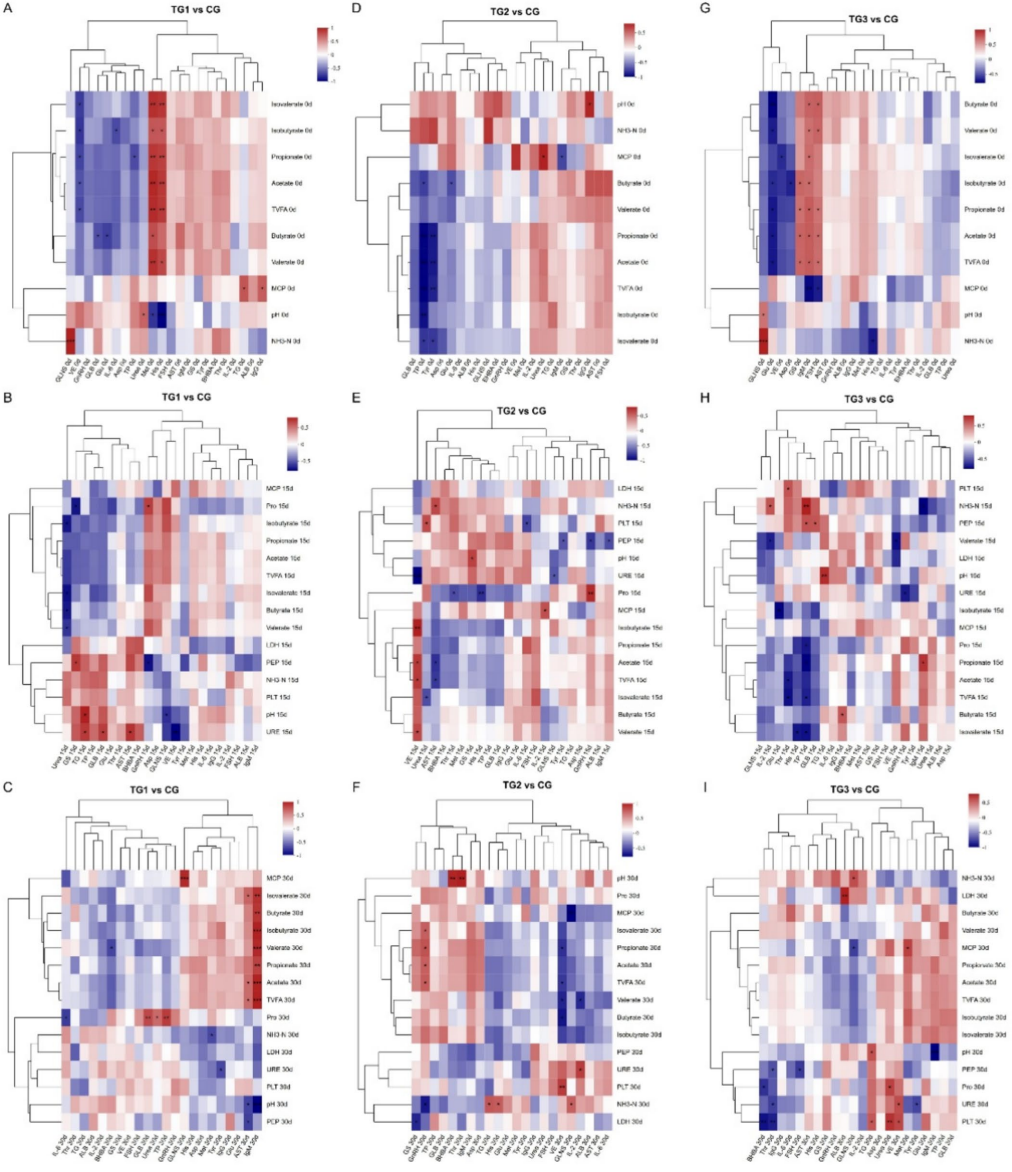
Correlation analysis of rumen fermentation parameters and serum indicators in three groups at different time. 0d: **(A-C)**; PP15d: **(D-F)**; PP30d: **(G-I)**.

In TG1, serum IgG at 0 d showed a significant positive correlation with MCP and a significant negative correlation with IL-6 and VFA (Figure 22A, *p* < 0.05). In TG2, MCP showed a significant positive correlation with URE and a significant negative correlation with IgM (Figure 22B, *p* < 0.05). In TG3, IgM and FSH were significantly positively correlated with propionate, acetate, and TVFA (Figure 22C, *p* < 0.05). At PP15 d, GnRH in TG1 was significantly positively correlated with Pro, while urea was significantly negatively correlated with Pro (Figure 22D, *p* < 0.05); In the TG2, VE was extremely significantly or significantly positively correlated with isobutyrate, acetate, and valerate (*p* < 0.01 or *p* < 0.05), while AST was significantly negatively correlated with TVFA (Figure 22E, *p* < 0.05). In TG3, IL-2 and TP were extremely significantly or significantly positively correlated with NH3-N (Figure 22F, *p* < 0.01 or *p* < 0.05). At PP30 d, IgM in TG1 was extremely significantly or significantly positively correlated with butyrate, isobutyrate, and valerate (Figure 22G, *p* < 0.01 or *p* < 0.05). In the TG2, GnRH was significantly positively correlated with isovalerate, propionate, and acetate (Figure 22H, *p* < 0.05); in the TG3, Pro and PLT were extremely significantly or significantly positively correlated with urea, and Pro and PLT were extremely significantly or significantly negatively correlated with BHBA (Figure 22I, *p* < 0.01 or *p* < 0.05).

#### 3.4.3 Correlation analysis between serum markers and nutrient utilization efficiency

**Figure 23:**
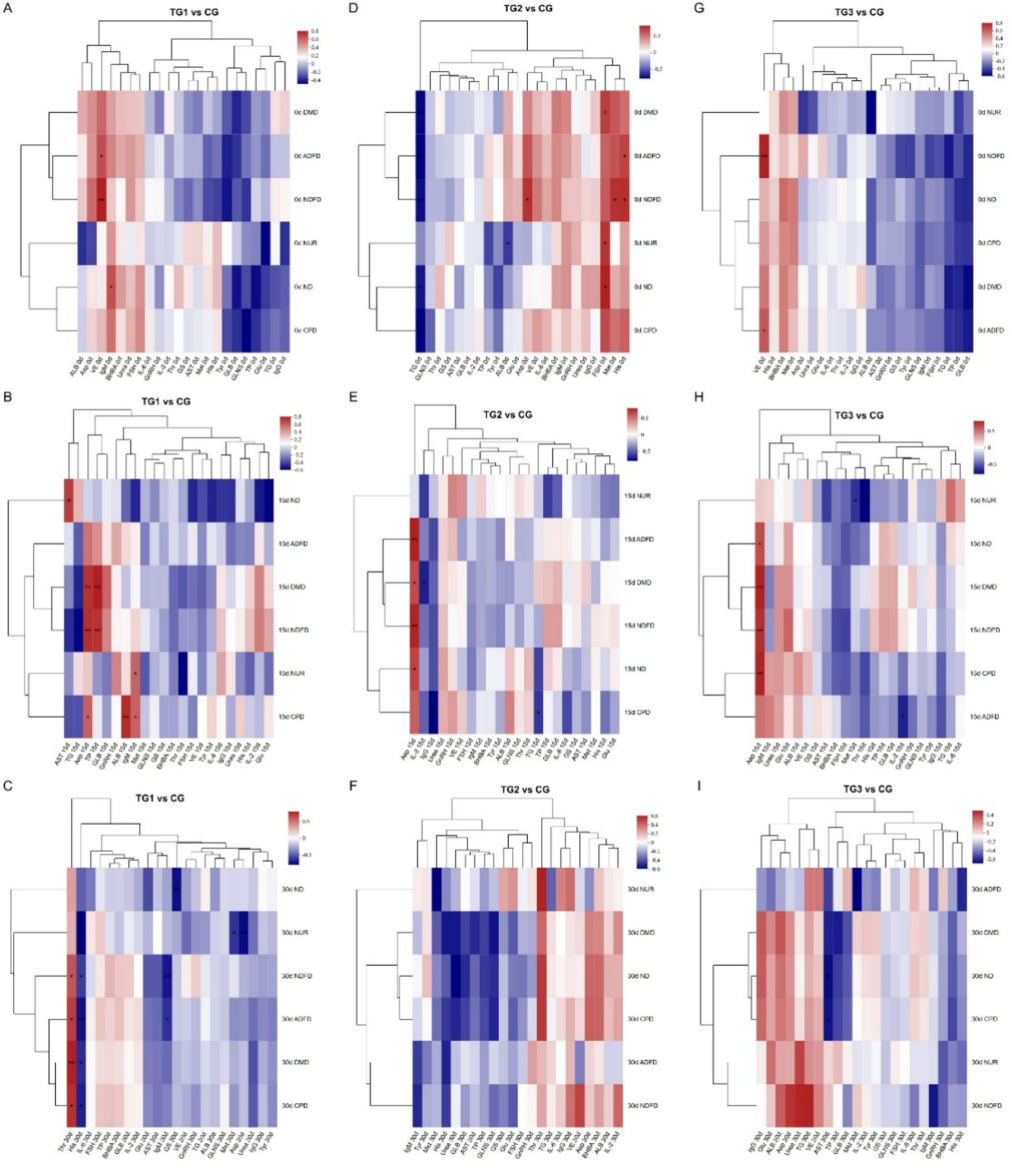
Correlation Analysis of Serum Markers and Nutrient Utilization Efficiency in three groups at different time. 0d: **(A-C)**; PP15d: **(D-F)**; PP30d: **(G-I)**.

In the TG1, serum VE at 0 d showed a highly significant or significant positive correlation with ADFD and NDFD (Figure 23A, *p* < 0.01 or *p* < 0.05); In the TG2, FSH was significantly positively correlated with NUR, ND, and DMD (Figure 23B, *p* < 0.05); The VE of TG3 was extremely significantly positively correlated with NDFD and others (Figure 23C, *p* < 0.01). At PP15 d, serum IgM in TG1 was significantly positively correlated with CPD and NUR (Figure 23D, *p* < 0.05); In the TG2, Asp was extremely significantly or significantly positively correlated with ADFD, DMD, NDFD, and ND, while IL-2 was significantly negatively correlated with DMD (Figure 23E, *p* < 0.01 or *p* < 0.05); Asp of TG3 was extremely significantly or significantly positively correlated with ND, DMD, NDFD, and CPD (Figure 23F, *p* < 0.01 or *p* < 0.05). At PP30 d, Thr of TG1 was extremely significantly or significantly positively correlated with DMD, CPD, NDFD, and ADFD, while IgM was extremely significantly or significantly negatively correlated with NDFD and ADFD (Figure 23G, *p* < 0.01 or *p* < 0.05). In the TG3, ND and CPD were significantly negatively correlated with AST (Figure 23I, *p* < 0.05).

#### 3.4.4 Correlation analysis of fecal and urinary metabolites with serum markers

**Figure 24:**
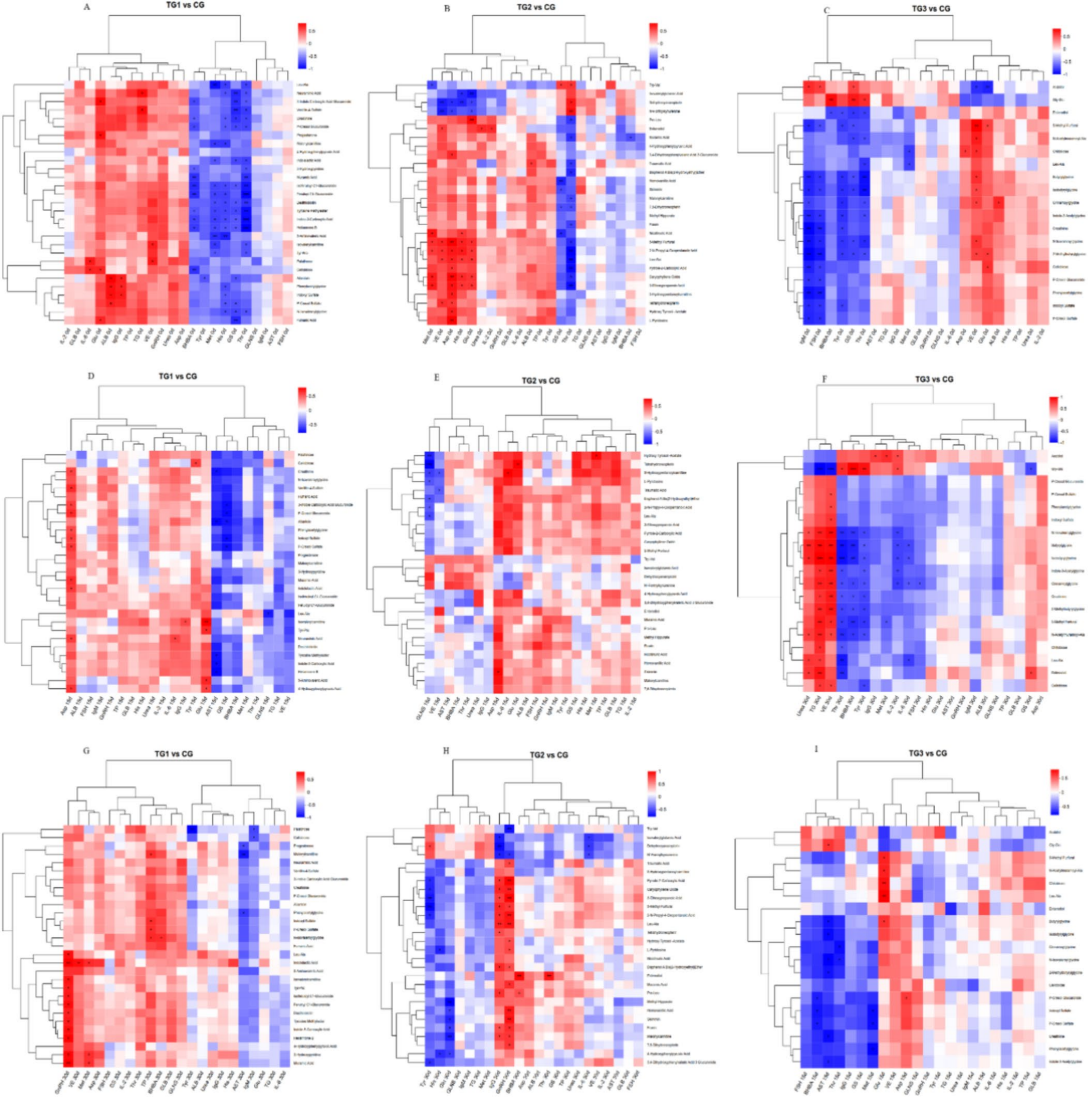
Correlation analysis of serum markers at three time points in the experimental group with fecal differential metabolites. 0d: **(A-C)**; PP15d: **(D-F)**; PP30d: **(G-I)**.

In the TG1 at 0 d, serum IL-6, IgG, and VE were significantly positively correlated with Palatinose, Indoxyl Sulfate, P-Cresol Sulfate, and others (*p* < 0.05); while BHBA, Tyr, and Met showed highly significant or significant negative correlations with Indole-3-carboxylic acid, Heliannone B, and others (Figure 24A, *p* < 0.01 or *p* < 0.05). VE of TG2 was highly or significantly positively correlated with caryophyllene oxide, traumatic acid, and urea, while IL-2 was highly or significantly positively correlated with enterodiol (Figure 24B, *p* < 0.01 or *p* < 0.05). In the TG3, IgM and FSH showed a highly significant or significant negative correlation with protein degradation products such as indoxyl sulfate, p-cresol sulfate, and p-cresol glucuronide, as well as with fibrosan and enterodiol (Figure 24C, *p* < 0.01 or *p* < 0.05).

At PP15 d, Asp of TG1 showed a highly significant or significant positive correlation with amino acid-derived toxins and microbial markers such as Cre, as well as with IL-6 and neuraminic acid; IgG showed a highly significant or significant positive correlation with isovalerylcarnitine (Figure 24D, *p* < 0.01 or *p* < 0.05); In the TG2, GLNS and VE were extremely significantly or significantly negatively correlated with markers of oxidative stress and amino acid metabolism, such as tetrahydroneopterin and 5-hydroxypentanoylcarnitine (Figure 24E, *p* < 0.01 or *p* < 0.05); In the TG3, urea, TG, and VE were highly significantly or significantly positively correlated with N-isovaleroylglycine (Figure 24F, *p* < 0.01 or *p* < 0.05).

At PP30 d, GnRH in the TG1 showed a highly significant or significant positive correlation with Leu-Ala, indolelactic acid, and heliannone B, while IgM showed a highly significant or significant negative correlation with carbohydrate-related metabolites such as cellobiose (Figure 24G, *p* < 0.01 or *p* < 0.05); In the TG2, IgG and GnRH were highly significantly or significantly positively correlated with 3-Ethoxypropanoic Acid, while GnRH was highly significantly or significantly negatively correlated with Trp-Val, N-Formylkynurenine, and IL-6, and IgG was highly significantly or significantly negatively correlated with Isovalerylglutamic Acid and Dehydrocyanopyrin (Figure 24H, *p* < 0.01 or *p* < 0.05); BHBA, AST, and Met of TG3 were significantly negatively correlated with Indoxyl Sulfate and P-Cresol Sulfate (Figure 24I, *p* < 0.05).

**Figure 25:**
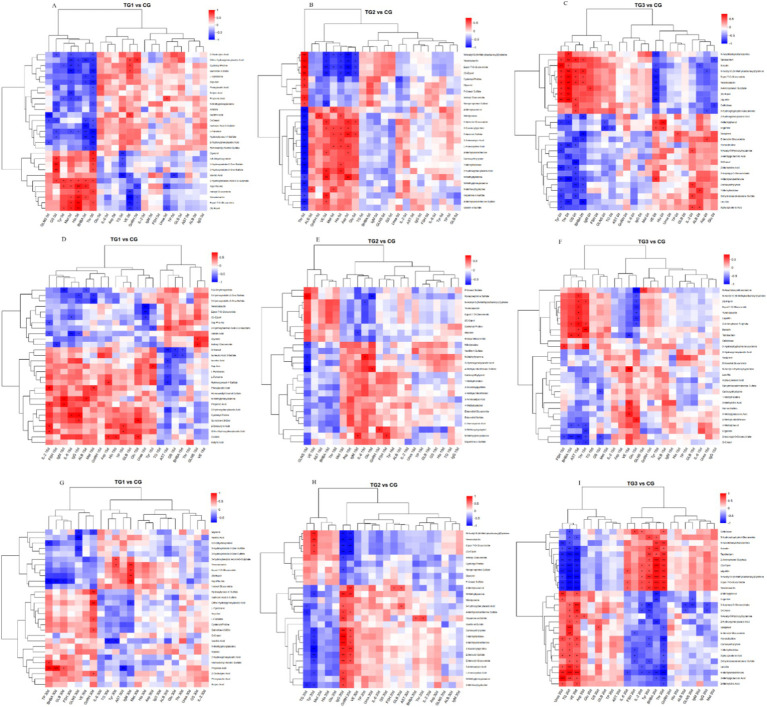
Correlation analysis of serum markers and urinary differential metabolites in the experimental group at three time points. 0d: **(A-C)**; PP15d: **(D-F)**; PP30d: **(G-I)**.

Compared with the CG, serum levels of GS, Tyr, Met, and BHBA in the TG1 at 0 d showed a highly significant or significant positive correlation with Yersiniabactin (Figure 25A, *p* < 0.01 or *p* < 0.05); In the TG2, Met, Asp, Thr and VE were significantly positively correlated with Caryophyllene Oxide, Traumatic Acid, Urea and IL-2, and with Enterodiol (*p* < 0.05). GS, Thr and BHBA were significantly negatively correlated with oxidative stress and microbial markers, etc. (Figure 25B); In the TG3, IgM was significantly positively correlated with 2-Aminophenol Sulphate (*p* < 0.05), while VE was extremely significantly or significantly negatively correlated with Baicalin, Equol 7-O-Glucuronide, and others (Figure 25C, *p* < 0.01 or *p* < 0.05).

At PP15 d, IL-2 and IgM in TG1 showed a significant positive correlation with propionic acid (*p* < 0.05), while urea and yersiniabactin showed a highly significant negative correlation (Figure 25D, *p* < 0.01); In the TG2, GLNS was significantly positively correlated with norepinephrine sulfate and significantly negatively correlated with nitrotyrosine and others (Figure 25E, *p* < 0.05); In The TG3, FSH and Thr were highly significantly or significantly positively correlated with 5-Hydroxytryptophol Glucuronide, and AST was highly significantly or significantly positively correlated with N-Acetyl-S-(N-Methylcarbamoyl)Cysteine; BHBA, AST, and Thr were highly significantly or significantly negatively correlated with 4-Methylphenol (Figure 25F, *p* < 0.01 or *p* < 0.05).

At PP30 d, GnRH in the TG1 was significantly negatively correlated with Glycerol (Figure 25G, *p* < 0.05); In the TG2, IgG and GnRH were highly or significantly positively correlated with N-Methyltyramine, and highly or significantly negatively correlated with Yersiniabactin and (S)-Equol (Figure 25H, *p* < 0.01 or *p* < 0.05); In the TG3, IL-6 showed a highly significant or significant positive correlation with liquiritin, while IL-2, Tyr, BHBA, and Thr showed a highly significant or significant positive correlation with (S)-equol; TG showed a highly significant or significant negative correlation with baicalin, and GnRH showed a highly significant or significant negative correlation with glycerol (Figure 25I, *p* < 0.01 or *p* < 0.05).

#### 3.4.5 Correlation analysis between fecal differential metabolites and nutrient utilization

**Figure 26:**
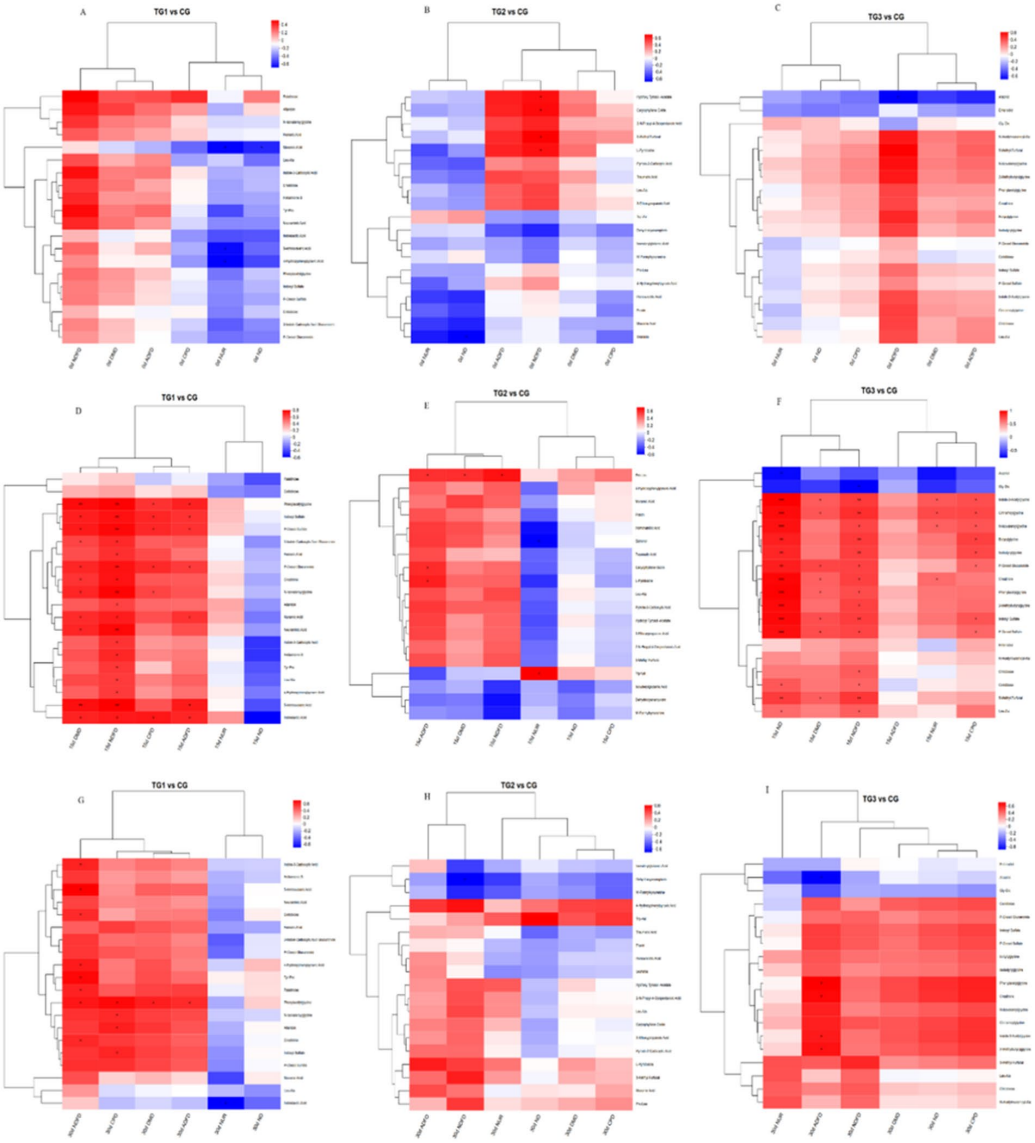
Correlation analysis of the efficiency of nutrient utilization and fecal differential metabolites in the experimental group at three time points. 0d: **(A-C)**; PP15d: **(D-F)**; PP30d: **(G-I)**.

In the TG1, muramic acid at 0 d showed a significant negative correlation with NUR and ND, while 5-aminovaleric acid and 4-hydroxyphenylpyruvic acid showed a significant negative correlation with NUR (Figure 26A, *p* < 0.05). Hydroxytyrosol of TG2 acetate and 5-methyl furfural showed a significant positive correlation with NDFD (Figure 26B, *p* < 0.05). In the TG3, Arabitol showed a significant negative correlation with NDFD (Figure 26C, *p* < 0.05).

In the TG1 at PP15 d, Phenylacetylglycine, Indoxyl Sulfate, and p-Cresol Sulfate showed a highly significant or significant positive correlation with NDFD and DMD (Figure 26D, *p* < 0.01 or *p* < 0.05). In the TG2, Pro-Leu was significantly positively correlated with ADFD, DMD, and NDFD; Caryophyllene Oxide and L-Pyridosine were significantly positively correlated with ADFD; and Skimmin was significantly negatively correlated with NUR (*p* < 0.05) (Figure 26E). In the TG3, Indole-3-Acetylglycine and others showed a highly significant or significant positive correlation with NDFD and DMD (*p* < 0.01 or *p* < 0.05), while Arabitol showed a significant negative correlation with ND, and Gly-Glu showed a significant negative correlation with NDFD (Figure 26F, *p* < 0.05).

At PP30 d, phenylacetylglycine in the TG1 was significantly positively correlated with NDFD, CPD, DMD, and ADFD, while indolelactic acid was significantly negatively correlated with NUR (Figure 26G, *p* < 0.05). In the TG2, dehydrocyanoprocine was significantly negatively correlated with NDFD (Figure 26H, *p* < 0.05). In the TG3, phenylacetylglycine, Cre, and others were significantly positively correlated with ADFD and significantly negatively correlated with Arabitol (Figure 26I, *p* < 0.05).

#### 3.4.6 Screening of key molecules based on network centrality

**Figure 27:**
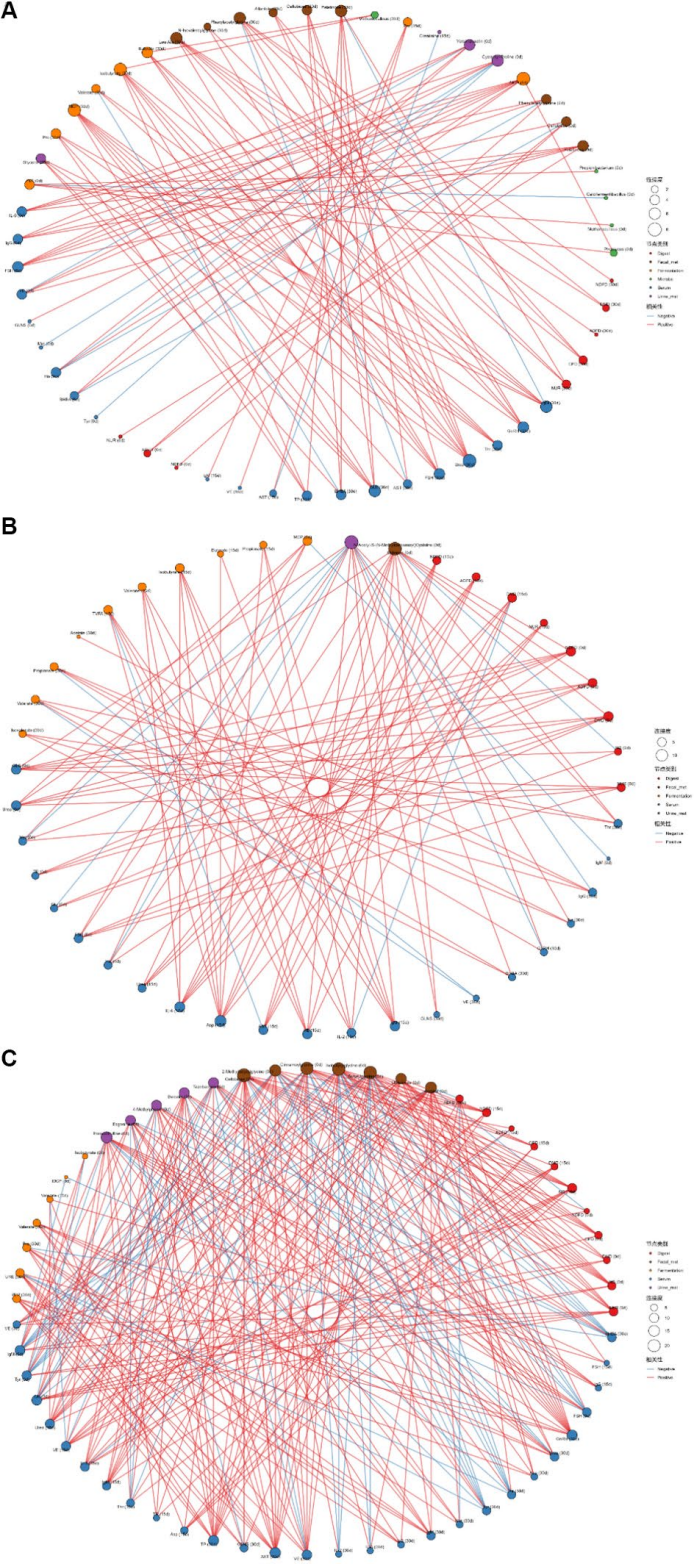
Core molecule interaction network. **(A)** TG1 vs CG; **(B)** TG2 vs CG; **(C)** TG3 vs CG.

An association network was constructed based on core nodes in each group (*p* < 0.05, |r| > 0.6). In the TG1, MCP and Urea served as core hubs (Degree ≥ 8), while protein fermentation products such as Isobutyrate and Phenylacetylglycine occupied secondary core positions, showing significant correlations with inflammation, immunity, reproduction, and energy metabolism (Figures 27A). In the TG2, Skimmin and Asp (Degree ≥ 9) served as core hubs. Skimmin was broadly associated with digestibility, nitrogen metabolism indicators, and immune and reproductive hormones. Asp was highly associated with short-chain fatty acids, inflammatory factors, and immunoglobulins (Figure 27B). The TG3 centers on Cellobiose (Degree = 24) and various glycine conjugates (Butyrylglycine, Isobutyrylglycine, Cinnamoylglycine, 2-Methylbutyrylglycine, Degree ≥ 17) as core hubs, showing significant correlations with reproductive hormones and immunoglobulins (Figures 27C).

## 4 Discussion

### 4.1 Protein sources influence rumen fermentation through microbial enrichment and metabolism

The rumen serves as the pivotal hub for nutrient conversion in ruminants, where fermentation processes, protein degradation, and microbial community structure and function are all influenced by dietary protein sources. Different protein sources can affect VFA production and nutrient digestion and utilization by regulating rumen microbial communities (Ran et al., 2024). Pro are key agents in rumen protein degradation and peptide conversion, with their activity directly influencing the absorption and utilization of dietary protein. This process is differentially regulated by microbial functional genes.

The rumen serves as the hub of nutrient metabolism in ruminants; its fermentation processes, protein degradation, and the structure and function of the microbial community are all influenced by the dietary protein source (Ran et al., 2024; Delosière et al., 2025). This study found that different protein sources regulate rumen fermentation by altering the abundance of microbial groups such as *Fibrobacter* and *Akkermansia* and the activity of related metabolic pathways (e.g., amino acid synthesis and secondary metabolite synthesis), but did not significantly alter the overall composition and diversity of the microbial community. In the TG1, *Methanomicrobium* was significantly negatively correlated with PP30 d butyrate and isovalerate levels; it may indirectly reduce the production of these two VFA through metabolic competition or by inhibiting the pathways for butyrate and isovalerate production. At the same time, propionate fermentation in the TG1 was higher at PP30 d than 0 d, indicating that the TG1 positively modulates the regulation of postpartum glucogenic precursors in cows over time, consistent with the findings of Paula (Paula et al., 2018). TG2 exhibited a significant increase in URE protein content at PP30 d, which promotes the conversion of non-protein nitrogen into NH₃-N, thereby providing available nitrogen for rumen microorganisms (Tao et al., 2022). In the TG3, the protein content of key nitrogen metabolism enzymes such as PP30 d Pro and URE significantly increased, accompanied by a relative rise in PEP activity. This indicates that proteins in the TG3 may be more susceptible to hydrolysis, promoting urea cycling and thereby altering nitrogen metabolism pathways (Liu et al., 2022). TG1 is dominated by the dual species *Caudoviricetes* and *Ca. Colisoma*. The microbial community fails to form an efficient and stable nitrogen metabolism network, with protein output pathways significantly downregulated, leading to reduced nitrogen deposition potential (Kong et al., 2010). TG2 centered on *Prevotella*, formed an efficient microbial nitrogen metabolism network, exhibiting effects most similar to the CG and demonstrating a positive impact on nitrogen utilization (Pope et al., 2010). TG3 enrichment in methanogenic archaea such as *Ca. Methanospirare* may alter carbon and nitrogen flux, leading to significant downregulation of AA biosynthesis and peptidoglycan synthesis pathways, thereby inhibiting microbial nitrogen assimilation and cell synthesis processes (Ungerfeld, 2020).

### 4.2 Protein source regulates serum markers and nutrient digestion and utilization

Based on the aforementioned changes in ruminal fermentation, different protein sources further influence serum parameters and the digestibility and utilization efficiency of nutrients in dairy cows. Specifically, different protein sources exert mu ltifaceted regulatory effects on ruminant metabolism, influencing not only the synthesis of ruminal MCP (Fan et al., 2025) but also regulating the absorption of serum nutrients and nitrogen metabolism balance (Oliveira et al., 2026; Chapelain et al., 2025). In this experiment, the levels of PP15 d GLB and Urea decreased and recovered by PP30 d, reflecting the inhibitory effect of parturition stress on protein anabolism and subsequent metabolic recovery. Postpartum levels of ALB, Cre, and blood ammonia in cows continued to decline, indicating a gradual reduction in metabolic load (Kuhla, 2020). Decreased GLNS and GS protein levels, coupled with elevated AST in the TG3, suggest that calving may temporarily increase the liver’s AA processing burden (Leoni et al., 2022). Due to the lack of significant grouping and interaction effects, different protein sources did not result in fundamental alterations in the overall AA oxidation and catabolism of the organism. The TG3 exhibited significantly higher TG content than other groups, consistent with GH levels peaking at PP15 d and declining by PP30 d, alongside elevated INS levels. This shift reflects a transition in the metabolic focus of cows from body fat mobilization to energy balance and anabolic metabolism (Sordillo, 2018). The V-shaped elevation and decline of IL-2 and IL-6 suggest that TG1 and TG2 may exert positive effects on regulating inflammatory responses (Spaans et al., 2022). In all groups, LH levels continued to rise, GnRH levels increased significantly, and E levels tended toward equilibrium following a sharp postpartum decline. Previous studies have shown that hypothalamic glial cells are involved in the regulation of reproductive neural circuits (Phillips et al., 2025), providing a foundation for understanding how the hypothalamus regulates reproductive function. Based on this, it is speculated that the dynamic changes in postpartum hormone levels observed in this study may reflect the gradual recovery of hypothalamic, pituitary, and ovarian function; however, the specific mechanisms in ruminants require further investigation. FSH levels in the TG3 were significantly lower than those in the TG1, while GnRH levels at PP30 d in the TG2 were significantly higher than those in the CG and TG3. Different protein sources may modulate hypothalamic and pituitary function, differentially regulating the secretion patterns of gonadotropins. Similarly, Ciccioli et al. demonstrated that postpartum nutritional status can significantly influence the recovery of endocrine function and reproductive performance in first-calf heifers (Ciccioli et al., 2003). Serum Met, Leu, and Phe levels showed significant interactions with protein source and time. The TG2 exhibited significantly higher 0 d Met, Tyr, and His levels compared to the TG1, indicating that the AA composition and digestibility/absorption characteristics of the TG2 may be superior to those of the TG1. This suggests enhanced hepatic metabolism and relatively greater AA utilization by other tissues. The TG2 demonstrated positive effects on IgG, IL-2, and partial serum AA supply. This may be attributed to the TG2 richness in *ω*-3 polyunsaturated fatty acids, which exert beneficial effects on anti-inflammation and immune modulation, thereby enhancing immune function and maintaining metabolic homeostasis in cows (Kra et al., 2023).

### 4.3 Analysis of fecal metabolites reveals protein sources’ impact on gut and systemic metabolism

To further investigate the effects of ruminal metabolic changes on the downstream metabolism of dairy cows, this study analyzed the metabolomes of feces and urine to elucidate the regulatory roles of different protein sources on intestinal and systemic metabolism, respectively.

#### 4.3.1 Fecal metabolites reveal close association between protein sources and gut metabolism

As end products of ruminal and intestinal metabolism, fecal metabolites directly reflect the differential regulatory effects of different protein sources on intestinal metabolism in dairy cows. Therefore, analyzing fecal metabolites can reveal the close association between protein sources and intestinal metabolic processes (Huyan et al., 2024; Jiang et al., 2024), providing a theoretical basis for elucidating their regulatory roles. The metabolome of the TG1 in this study showed distinct separation from the CG. This may be attributed to the TG1 richness in antinutritional factors such as glucosinolates, sinigrin, and tannins. These compounds regulate gut microbiota structure, influence bile secretion, or serve as precursors converted by microorganisms into specific metabolites, thereby altering the hindgut metabolic environment (Szajewska et al., 2025). Enrichment in pathways such as riboflavin metabolism provides direct evidence for this mechanism (Magnúsdóttir et al., 2015). The enrichment of rumen methanogens in TG3 can compete for carbon sources required for nitrogen assimilation (Fitzsimons et al., 2013), affecting AA biosynthesis, bile secretion, and steroid hormone synthesis, which is similar to the findings reported by Ungerfeld (Ungerfeld, 2020). The rumen microbial structure and function of the TG2 were similar to those of the CG, supporting cows to develop favorable metabolic adaptability and achieving the lowest metabolic cost to facilitate a smooth transition through the peripartum period.

#### 4.3.2 Urine metabolites reflect intrinsic connections between protein sources and systemic metabolism

Different protein sources can alter rumen microbiota function and metabolites, and after integration in the liver, they influence nitrogen utilization (Fan et al., 2025; Prahl et al., 2022); differences in urinary metabolite profiles reflect this systemic regulation and can serve as biomarkers for evaluating the nutritional and physiological effects of protein sources (Bertram et al., 2011); Unlike fecal metabolites, urinary metabolites primarily reflect the systemic metabolic status of dairy cows and can further reveal the indirect effects of protein sources on systemic physiology. In this study, significant metabolic differences were observed between the CG and TG1. with differential pathways including nutrient digestion and absorption, AA metabolism, phenylalanine metabolism, and nicotinic acid and nicotinamide metabolism. This indicates that the TG1 not only affects nitrogen utilization in co ws (Hulshof et al., 2017), but may also influence relevant metabolic enzymes and regulatory signals through other bioactive components such as sinigrin derivatives (Cory et al., 2018), thus triggering metabolic regulation in cows across multiple metabolic dimensions. TG2 may play a positive role in efficient nitrogen utilization and recycling through fine-tuning the metabolism of Try, Tyr, and branched-chain amino acids (Roth et al., 2021). The TG3 showed significant enrichment in pathways such as unsaturated fatty acid synthesis and bile secretion, consistent with liver and bile load signals detected in feces and serum. This indicates that the TG3 may influence lipid metabolism balance in cows.

### 4.4 Multi-indicator correlation analysis reveals metabolic regulation of protein sources

To integrate the complex relationships among rumen microbiota, fermentation parameters, serum markers, and fecal and urinary metabolites, a multivariate association analysis was conducted to explore the potential pathways through which protein sources influence postpartum physiology in dairy cows via changes in rumen and intestinal metabolism and serum markers, focusing on three aspects: digestibility, immune response, and reproductive hormones.

#### 4.4.1 Intestinal metabolism and digestibility

Correlation analysis first revealed a close relationship between rumen fermentation parameters and metabolites and nutrient digestibility in dairy cows. Dietary protein sources can influence apparent nutrient digestibility by modulating the structure of the gut microbiota, thereby altering nutrient degradation pathways and the abundance of metabolites (Rivera-Chacon et al., 2024). In this process, differentially expressed metabolites serve as a central hub for analyzing the association between dietary protein sources and apparent digestibility and nitrogen utilization in dairy cows (Cai et al., 2025). In the TG1, various amino acids including Thr, His, and Met were significantly correlated with NDFD, DMD, and ADFD, revealing that amino acid metabolism and fiber digestibility play a key role in regulating nutrient utilization from protein sources. In feces, muramic acid, 5-aminovaleric acid, and 4-hydroxyphenylpyruvic acid are associated with cell wall degradation, amino acid metabolism, and protein fermentation, respectively, and show a significant negative correlation with NUR, suggesting that nitrogen utilization in dairy cows may be limited (Du et al., 2023). At PP15 d and PP30 d, microbial fermentation products of proteins and AA including phenylacetylglycine, indoxyl sulfate, and p-cresol sulfate exhibited significant positive correlations with NDFD and DMD. This indicates incomplete digestion and absorption of proteins in the foregut, followed by substantial microbial degradation in the hindgut, potentially leading to increased metabolic burden (Evenepoel et al., 2009). Hydroxytyrosol-Acetate and 5-Methyl Furfural in TG2 0 d feces showed significant positive correlations with NDFD. Hydroxytyrosol-Acetate and other compounds can regulate rumen antioxidant activity, thereby playing a positive role in maintaining the activity of fiber-degrading bacteria (Zou et al., 2023; Baldi et al., 2023). At PP15 d, Pro-Leu, Caryophyllene Oxide, and L-Pyridine remained positively correlated with ADFD and DMD. This may be attributed to the presence of viscous polysaccharides and *α*-linolenic acid in the TG2, which synergistically improved the microbial metabolic environment and enhanced antioxidant activity (Baldi et al., 2023; Deters and Hansen, 2019), thereby promoting fiber degradation and efficient nutrient utilization. TG3 fecal 0 d arabitol, as a poorly absorbed carbohydrate, showed a significant negative correlation with NDFD, indicating that carbohydrate metabolism may be incomplete. At PP15 d, Indole-3-Acetylglycine showed positive correlations with NDFD and DMD. This pattern of association between the metabolite and digestibility aligns with the interaction dynamics between the rumen microbiome and metabolome under dietary regulation (Du et al., 2023). The negative correlations between Arabitol and ND, Gly-Glu and NDFD, along with the positive correlations between PP30 d Phenylacetylglycine, Cre and ADFD, indicate that lignified fiber and phytate may be present in the TG3 diet, potentially affecting nutrient release and microbial utilization. This finding aligns with Brito research on the regulatory effects of different protein sources on nutrient utilization in cows (Brito and Broderick, 2007).

#### 4.4.2 Metabolites and immune response

Metabolites of feces and urine are closely associated with the gut microbiota and immune response, collectively forming the body’s immune regulatory network (Castillo-Lopez et al., 2024). At the same time, metabolic products from the rumen and intestines can enter the circulatory system, where they interact with serum immune markers, thereby influencing the establishment and recovery of immune function in breeding cows. Indoxyl sulfate and p-cresol sulfate are characteristic metabolites of gut microbial metabolism (Lin et al., 2022). These metabolites showed significant positive correlations with IL-6, IgG, and VE in the serum of cows in the TG1 at 0 d, suggesting that such microbial-derived substances may participate in regulating the immune status of cows during the perinatal period. At PP15 d, IL-6 showed a positive correlation with neuraminic acid, while aspartate and Cre exhibited positive correlations with toxins. This indicates that the intestinal metabolic environment in the TG1 is closely linked to the sustained low-grade inflammatory state in cows (Calder, 2013; Zhang et al., 2025). Enterodiol is a core metabolite produced by rumen microbial metabolism of plant lignans (Landete, 2012). Its positive correlation with 0 d Urea and IL-2 in the TG2 indicates efficient synergistic interaction between immune activation and nitrogen metabolism. Significant positive correlations were observed between 0 d VE and Caryophyllene Oxide, Traumatic Acid, Urea, and IL-2 with Enterodiol, while negative correlations were noted between PP30 d IL-6, IgG and Isovalerylglutamic Acid, Dehydrocyanaropicrin, among others. These primarily stem from VE’s antioxidant and immunomodulatory effects. VE can alleviate inflammation induced by oxidative stress during the peripartum period in cows by enhancing the host antioxidant defense system, thereby jointly maintaining metabolic health and reproductive recovery during this critical phase (Deters and Hansen, 2019). Serum IgM and FSH levels in TG3 0 d were significantly positively correlated with fecal Indoxyl Sulfate, P-cresol sulfate, P-cresol glucuronide, and other protein putrefaction products, as well as cellobiose. Among these, indoxyl sulfate and P-cresol sulfate are characteristic products of gut microbial metabolism, whose entry into the circulatory system depends on intestinal barrier permeability (Lin et al., 2022). FSH levels at PP15 d were positively correlated with urinary 5-hydroxytryptophol glucuronide, AST, and N-acetyl-S-(N-methylcarbamoyl) cysteine, among other toxins. These correlations align with the pattern of effects observed when gut-derived microbial toxins enter the bloodstream through a compromised intestinal barrier, subsequently influencing reproductive hormones and liver function markers (Evenepoel et al., 2009). This suggests that the TG3 diet may compromise intestinal integrity in cows, allowing endogenous toxins to enter the circulation and thereby disrupting immune system regulation and reproductive hormone control (Calder, 2013; Zhang et al., 2025).

#### 4.4.3 Metabolites and reproductive hormones

In addition, specific metabolites can regulate the secretion of reproductive hormones by activating key signaling pathways; they serve as biomarkers reflecting the relationship between dietary protein sources and the endocrine system of dairy cows (Wang et al., 2023), thereby providing a basis for elucidating the regulatory relationship between metabolites and reproductive hormones. This provides crucial evidence for elucidating the regulatory mechanisms between metabolites and reproductive hormones. In this study, serum BHBA, Tyr, Met, and other metabolites in the TG1 at 0 d were negatively correlated with fecal metabolites such as Indole-3-Carboxylic Acid and Heliannone B, indicating metabolic stress in cows. This finding aligns with previous research showing that elevated BHBA levels during the peripartum period impair reproductive function (Bicalho et al., 2017). The association with Heliannone B reflects the synergistic interaction between metabolic stress and oxidative stress (Sordillo et al., 2009). The positive correlation between GnRH at PP30 d and fecal Leu-Ala, indoleacetic acid, and Heliannone B, along with the negative correlation with urinary glycerol, indicates that GnRH secretion is influenced by protein metabolism disorders and oxidative stress. TG2 showed significant positive correlations between GnRH and isovalerate, propionate, acetate, etc., at PP30 d, while exhibiting negative correlations with Trp-Val and N-formylkynurenine in the tryptophan metabolic pathway. This indicates positive synergistic interactions among energy supply, antioxidant environment, and reproductive signaling. Propionate serves as a key glucogenic precursor, providing energy for reproductive activities and establishing a high-energy, low-inflammation metabolic homeostasis. This creates favorable conditions for the recovery of reproductive performance (Ziegler et al., 2025). GnRH is positively correlated with antioxidants and negatively correlated with inflammatory metabolites; a metabolic environment with a low inflammatory burden may promote GnRH secretion and play a positive role in the recovery of postpartum reproductive performance (Gupta et al., 2024; Castillo-Lopez et al., 2025). TG3 FSH 0 d showed negative correlations with indoxyl sulfate, p-cresol sulfate, and cellobiose, consistent with findings by Zhou (Zhou et al., 2024). The accumulation of toxins such as indoxyl sulfate may disrupt reproductive function. FSH levels at PP15 d were positively correlated with the serotonin metabolite 5-hydroxytryptophan glucuronide. A study by Wang et al. (2025) indicated that bidirectional regulation between the hypothalamus and the gut microbiota is critical for periparturient cows, and that disruption of this homeostasis may affect the hypothalamic, pituitary, and gonadal regulation of hormone secretion, such as FSH. This conclusion is consistent with the findings of the present study, which suggest that nutritional factors may influence the secretion of reproductive hormones through similar pathways. Furthermore, D'Occhio et al. (2019) noted that nutritional factors such as energy and protein levels can influence the recovery of postpartum reproductive function in beef cattle through metabolic hormone signaling, providing theoretical support for the present study.

#### 4.4.4 Limitations of the study

It should be noted that the metagenomic and metabolomic analyses in this study were based solely on samples collected on the 0 d and therefore do not reflect the dynamic changes in postpartum microbiota and metabolites. Consequently, the associations between the 0 d omics data in Section 3.4 and the physiological parameters measured on the 0 d, PP15 d and PP30 d represent temporal correlations and do not imply causality. Future studies should incorporate longitudinal multi-omics data to further validate the findings of this study.

## 5 Conclusion

**Figure 28:**
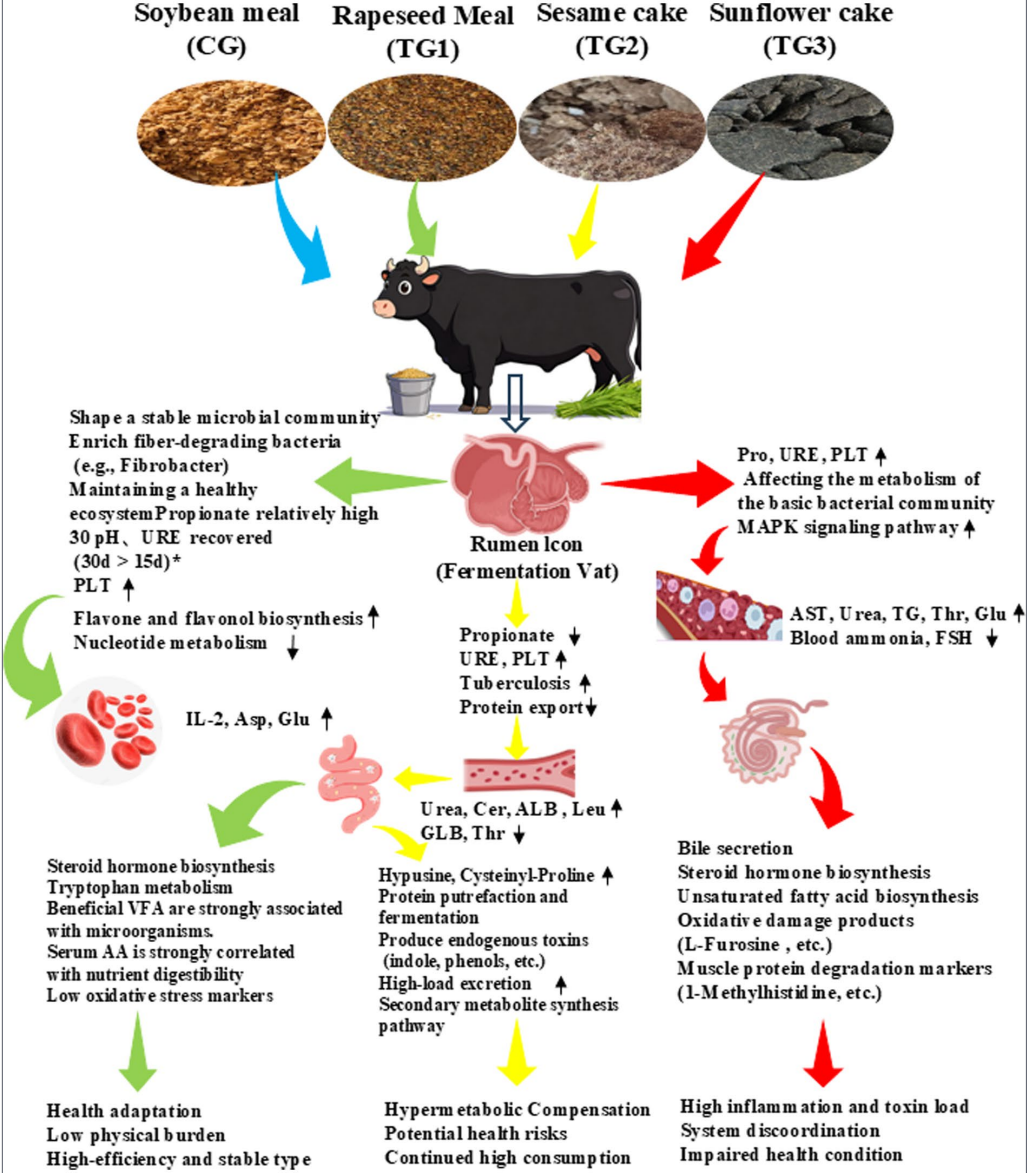
Schematic diagram of the main findings of this study.

The main findings of this study are summarized in Figure 28. The rapeseed meal group caused excessive protein fermentation, producing endogenous microbial toxins that triggered high oxidative stress and metabolic burden. The sunflower cake group had a lesser impact on conventional metabolic indicators, but its association with reproductive hormones, intestinal toxins, and permeability markers impaired reproductive performance. The linseed cake group maintained rumen fermentation and intestinal metabolism in cows, demonstrating superior effects compared to both rapeseed meal and sunflower cake groups. It serves as a high-quality protein source for replacing soybean meal in diets for periparturient breeding cows, providing theoretical support for designing rations for cows during this physiological stage.

## Data Availability

The datasets presented in this study can be found in online repositories. The names of the repository/repositories and accession number(s) can be found at: https://www.ncbi.nlm.nih.gov/, PRJNA1436962.
